# Evolved populations of Listeria monocytogenes related to biofilm formation and biocide stress in the context of food production environment niches

**DOI:** 10.1099/mgen.0.001611

**Published:** 2026-01-21

**Authors:** Oleksii Omelchenko, Ana Victoria Gutiérrez, Maria Diaz, Erin Lewis, Maria Solsona Gaya, Mark A Webber, Matthew Gilmour

**Affiliations:** 1 Quadram Institute Bioscience, Norwich, UK; 2 University of East Anglia, Norwich, UK; 3 Food Standards Agency, London, UK; 4 Center For Microbial Interactions, Norwich, UK

**Keywords:** adaptive evolution, benzalkonium chloride, biofilm, disinfectant, food processing environment, *Listeria monocytogenes*, reduced susceptibility

## Abstract

Cleaning and disinfection of food production environments (FPE) are fundamental components of food safety programmes designed to control microbial pathogens and prevent food contamination. Yet, FPE can still harbour foodborne pathogens, including *Listeria monocytogenes,* a significant concern to food manufacturers and health authorities due to the high mortality rate associated with invasive listeriosis. Mechanisms contributing to *L. monocytogenes* persistence in FPE include biofilm formation and reduced susceptibility to biocides, such as benzalkonium chloride (BC), for which several mechanisms are known. We hypothesized that prolonged exposure to disinfectants and other FPE-associated stressors would drive *L. monocytogenes* adaptation, resulting in the accumulation of genetic mutations linked to biofilm formation and reduced biocide susceptibility. To test this, we developed a biofilm persistence model, which studied 30 consecutive passages of biofilm-associated cells grown on stainless steel under sub-inhibitory BC concentrations. Whole-genome sequencing of evolved populations identified mutations that were associated with biofilm lineages and/or BC exposure. Non-synonymous mutations were identified in genes and pathways involved in metal homeostasis, stress response and pyrimidine biosynthesis. In addition, reduced susceptibility to BC arose through multiple independent mutations within the *fepRA* operon, encoding FepR transcriptional repressor and FepA MATE efflux pump. These mutations were observed across both planktonic and biofilm lifestyles, resulting in a comparable level of reduced susceptibility to BC in both states. Several loci with fixed mutations associated with biofilm lineages were identified, including the *ykoK* riboswitch leader, the pyrimidine synthesis operon and the stress response-related gene *rsbU*. Collectively, these findings provide new insights into the genetic mechanisms underlying *L. monocytogenes* biofilm persistence and reduced biocide susceptibility in the context of FPE and reveal novel targets potentially exploited by *L. monocytogenes* to establish and maintain niches in unfavourable environments.

**Impact Statement**

This study advances the understanding of *Listeria monocytogenes* adaptation to food processing environments, with a focus on sequence type 121, a prevalent lineage commonly associated with contamination in food and food processing facilities. Key findings include the identification of novel pathways linked to reduced disinfectant susceptibility and biofilm persistence, which may be exploited by *L. monocytogenes* to establish and maintain niches in otherwise unfavourable conditions. These pathways encompass mechanisms related to metal ion homeostasis, *de novo* pyrimidine synthesis and general stress response. Additionally, putative compensatory mechanisms were detected for some of the described pathways at later evolutionary stages, insights that would not be captured through single-time-point studies, highlighting the necessity of a longitudinal approach. We also expanded current knowledge of *L. monocytogenes* reduced susceptibility to benzalkonium chloride through the *fepRA* operon. This included the discovery of previously unreported mutations in the untranslated region of *fepR*, its coding sequence and in the *fepA* efflux pump. These findings provide molecular targets for surveillance of disinfectant-adapted strains in food processing environments and highlight the need for biocide management strategies that limit the selection of efflux-based tolerance mechanisms. Ultimately, understanding how *L. monocytogenes* adapts to food processing environments enables the development of improved food safety measures. These insights can support more effective strategies to prevent contamination of food production facilities and reduce the risk of in-factory foodborne pathogen transmission.

## Data Summary

Data are available in the National Center for Biotechnology Information under BioProject: PRJNA1276474 (Quadram Institute Bioscience). SRA and BioSample accession numbers are provided in Table S3. The authors confirm that all supporting data and protocols have been provided within the article or through supplementary data files.

## Introduction

Food production environments (FPE) are complex ecosystems that can harbour diverse microbial communities capable of surviving despite intensive microbiological control procedures. These communities are shaped by multiple factors that promote the establishment of microbial niches and select for specific microbial members [1,2]. While most of FPE-associated communities are non-pathogenic, a smaller proportion may contain pathogenic bacteria, posing significant risks to food safety [3,4]. One pathogen of significant concern to both food manufacturers and public health authorities is *Listeria monocytogenes*, the causative agent of invasive listeriosis. Although the incidence of listeriosis remains relatively low, ranging from 0.13 to 0.38 cases per 100,000 people in England and Wales in 2023, the consequences can be severe. Mortality was 21.6% in England and Wales in 2023, with fatal obstetrical outcomes occurring in 20–30% of affected pregnancies [5,9].

A defining trait of *L. monocytogenes* is its ability to persist on FPE surfaces within biofilms, self-organized microbial communities encased in a matrix of extracellular polymeric substances [10]. Once established, these biofilms are notoriously difficult to eradicate and can persist in food factories for decades, leading to recurrent contamination of food products [11]. *L. monocytogenes* biofilms commonly develop on surfaces, such as conveyor belts, drains, surface cracks and crevices [12], where they protect against multiple environmental stressors, including physical (shear-force, reduced temperatures), chemical (bactericidal compounds) and biological (nutrient/niche competition) stressors [13,15].

Certain subtypes of *L. monocytogenes* are more commonly associated with FPEs, particularly lineage II strains [16,18] Among lineage II strains*,* sequence type 121 (ST121) is notable for its frequent isolation from FPE and food products, including dairy, fish, turkey and sandwiches, although it is rarely associated with clinical cases [11]. Despite a lower virulence potential [19], the persistence of ST121 in FPEs poses a critical food safety challenge. Notable incidents have occurred in the UK [20] and Norway [21], where contaminated ready-to-eat foods were consumed by immunocompromised individuals.

This persistence is facilitated by intrinsic and acquired genetic traits that enhance *L. monocytogenes* survival and niche establishment within larger microbial communities [22,24], under refrigeration temperatures, high salt concentrations, low pH and exposure to biocides [12,22, 25, 26]. Among the most widely used disinfectants in FPE is benzalkonium chloride (BC) [27], a broad-spectrum disinfectant with a multi-factorial mode of action, including the inactivation of metabolic enzymes, denaturation of proteins and disruption of membrane bilayers [28,29]. However, *L. monocytogenes* can develop reduced susceptibility to BC following prolonged exposure, which may also reduce susceptibility to antibiotics such as ciprofloxacin and gentamicin [30]. Known reduced susceptibility mechanisms to BC include several efflux pump systems, such as QacH encoded on the transposon Tn*6188* [31], EmrE on the genomic island LGI1 [32], two small multidrug resistance efflux pumps BcrB and BcrC located on a *bcrABC* cassette and the *fepRA* operon consisting of multidrug and toxic compound extrusion (MATE) efflux pump (FepA) and its local repressor (FepR) [27,33,36].

Complementing these reduced susceptibility mechanisms, *L. monocytogenes* also harbours a repertoire of biofilm-associated traits that support its persistence across varied environmental conditions [37]. These include surface structures such as flagellin A [encoded by *flaA* (*lmo0690*)], which facilitates initial attachment, and ActA, which promotes bacterial aggregation and biofilm development. Internalins A and B (InlA and InlB) also contribute to biofilm maturation by enhancing surface adhesion and cell-to-cell interactions [38]. Biofilm regulation is further influenced by quorum sensing systems, including the LuxS/AI-2 system, which has been associated with repression of biofilm-related genes, and the *AgrBDCA* operon, which promotes biofilm formation through peptide-based signalling [38,39].

We hypothesized that *L. monocytogenes* adapts to FPE-associated stressors through acquisition of genetic mutations that enhance biofilm formation and confer reduced biocide susceptibility. However, the specific genetic changes driving this adaptation, especially in the context of biofilm-associated cells, remain unknown. To address this, we developed a continuous *in vitro* biofilm model that resembles key conditions in FPE and allows us to study the long-term evolution of *L. monocytogenes* under sub-inhibitory BC exposure. We selected a biocide-susceptible, 1980s-era, chicken-derived *L. monocytogenes* ST121 isolate as a naive baseline strain. Being an older susceptible isolate, it is less likely to have undergone contemporary FPE bottlenecks, thus providing an opportunity to observe adaptation under experimentally imposed stresses. After 30 consecutive passages of evolution, followed by whole-genome sequencing of the evolved populations, we identified adaptive mutations that became fixed over time and described the genetic mechanisms driving *L. monocytogenes* persistence in food processing environments.

## Methods

### Bacterial strains and growth conditions

*L. monocytogenes* isolate BL87-028 was selected as the wild-type, naive strain for the biofilm persistence model (BPM) experiment. This strain was isolated in 1987 from a chicken in the UK. Based on the analysis of the complete hybrid genome assembly (BioSample: SAMN28229363), previously performed by our group using short- and long-read sequencing, we identified it as belonging to ST121 and as a non-carrier of *qacH* and *bcrABC* cassettes. This strain was selected primarily due to its susceptibility to BC, exhibiting a minimum inhibitory concentration (MIC) of 3.12 µg ml^−1^ – significantly lower than that of closely related strains [40].

### *In vitro* bead-based biofilm persistence model

An *in vitro* bead-based biofilm persistence model [41,42] was adapted to investigate the effects of exposure of *L. monocytogenes* to steady and ascending BC concentrations as well as to biofilm persistence (Fig.e S1, available in the online Supplementary Material; Supplementary Material 1). In the context of this study, we defined biofilm persistence as the ability of a microbial population to maintain long-term colonization of a surface through successive cycles of biofilm attachment and dispersal, under recurrent antimicrobial and/or environmental stress.

Four experimental conditions (biofilm steady-exposed, biofilm ladder-exposed, planktonic steady-exposed and planktonic ladder-exposed lineages) and two control conditions (biofilm non-exposed and planktonic non-exposed lineages) (Fig. S1) were performed, with four biologically independent lineages each.

For all conditions, the inoculum was prepared by growing *L. monocytogenes* in Brain Heart Infusion (BHI) broth (NutriSelect^®^, Millipore, Burlington, MA, USA) for 18 h at 37 °C, followed by dilution to an optical density of 0.05 AU (OD600_nm_) in 1/10 BHI broth. Independent lineages were established from different colonies.

For biofilm lineages, one bead per well was placed in a 12-well microtiter Costar plate (Corning, Corning, NY, USA), containing 2 ml of the diluted culture [0.05 AU (OD600_nm_) in 1/10 BHI broth] and incubated for 48 h at 20 °C under mild agitation (60 r.p.m.). After incubation, beads were individually collected and washed by submerging twice into sterile PBS to remove loosely attached cells before being transferred into a subsequent 12-well microtiter plate containing sterile 1/10 BHI broth and a sterile bead. At each subsequent passage, only the bead most recently exposed to *Listeria* was collected for transfer to the next culture. This process was repeated continuously for a total of 30 passages, equivalent to 562 generations, as calculated following the method described by Poltak and Cooper [41]. Stainless-steel (grade 316) beads with a diameter of 6 mm (surface area=113.1 mm^2^) (Simply Bearings Ltd, Leigh, Lancashire, UK) were used as the substrate for biofilm formation. To distinguish between the beads during passaging (i.e. to reliably collect only the bead most recently exposed to *Listeria*), alternating beads were marked with lacquer in a small area of the bead (≈5% of the total area), leaving the majority of the bead unmarked for the availability of the stainless-steel surface. Prior to use, the beads were rinsed with water, autoclaved and then submerged in 70% ethanol. Prior to use, the beads were air-dried for 5 min to allow complete evaporation of residual ethanol. All beads were used only once.

Planktonic lineages (without the bead) were established in parallel as controls for the biofilm condition. For planktonic lineages, 2 ml of the diluted culture (0.05 AU (OD600_nm_) in 1/10 BHI broth) was incubated without beads in a 12-well microtiter plate for 48 h at 20 °C under mild agitation (60 r.p.m.). After incubation, 30 serial passages at 48 h were performed by preparing a 1/100 dilution of the bacterial culture in sterile 1/10 BHI broth and incubating as described before.

For lineages exposed to the stressor BC (C8H17-C18H37 alkyl distribution, Sigma-Aldrich, St. Louis, MO, USA), two experimental models were tested. In the steady model, cultures were continuously passaged in 1/10 BHI broth supplemented with 0.625 µg ml^−1^ BC. In the ladder model, passage 11 of the steady model was split from the steady lineages and subjected to increasing BC concentrations: 1.3 µg ml^−1^ from passage 11 to 30 (1-step ladder) or, at passage 22, a further increase to 1.75 µg ml^−1^ until passage 30 (2-step ladder). Glycerol stocks were prepared at three selected time points – ‘early’ (passage 11), ‘mid’ (passage 22) and ‘late’ (passage 30), for both steady and ladder models. Biofilm beads were preserved in 1.5 ml of 20% glycerol prepared in 1/10 BHI broth, while 750 µl of planktonic cultures were mixed 1:1 with 50% glycerol. All samples were stored at −70 °C in 96-well deep-well plates (Sigma-Aldrich, St. Louis, MO, USA). For biofilm lineages, the stored beads were later processed by detaching bead-attached cells (see the ‘Cell attachment capacity’ section), which were then mixed with 50% glycerol (1: 1 ratio) and stored again at −70 °C.

At each time point, the entire culture obtained from a biofilm or planktonic lineage was considered a *population*, representing the collective population-level phenotype. Both detached biofilm and planktonic cultures were plated on BHI agar and incubated at 37 °C for 18 h. From each population lineage, three random colonies, hereafter referred to as *individual colonies*, representing clonal subpopulations, were isolated and preserved in 20% glycerol at −70 °C. These stocks were used for phenotypic characterization of both population-level and individual-colony traits, as well as for WGS of populations and individual colonies.

### Cell attachment capacity

The cell attachment capacity assay was used to quantify the population of the parental strain on beads during BPM optimization, as well as to measure the surface attachment capacity of late evolved lineages.

Following biofilm formation (see the ‘*In vitro* bead-based biofilm persistence model (BPM)’ section), beads were submerged twice in 1 ml of sterile PBS contained in a 24-deep well plate (Axygen, Corning, NY, USA) to remove loosely attached cells. Washed beads were transferred into 1 ml PBS and then sonicated at 50 Hz for 5 min to detach cells from the surface of the beads. Plate counts of the bead-detached cell suspensions were performed on BHI agar, incubated at 37 °C for 24 h, and the CFUs per bead were then calculated using the following formula: [(Number of colonies × dilution factor) / volume of culture plated] / volume used to sonicate one bead. Three biological and three technical replicates were included for each tested isolate.

### Biocide sensitivity testing

The MIC of BC was determined using a modification of the agar dilution antimicrobial susceptibility method [43]. A 96-well pin replicator (Boekel Scientific, Feasterville-Trevose, PA, USA) was used to inoculate 10^4^ c.f.u. ml^−1^ on lysed horse blood-Mueller–Hinton agar containing increasing BC concentrations (0 µg ml^−1^, 2.5 µg ml^−1^, 5 µg ml^−1^, 7.5 µg ml^−1^, 10 µg ml^−1^, 12.5 µg ml^−1^, 15 µg ml^−1^ and 20 µg ml^−1^). The growth-control plates (0 µg ml^−1^ BC concentration) were inoculated first and last to detect contamination or significant biocide carryover. MICs were recorded as the lowest BC concentration that entirely inhibited the growth of the strains. The mean MIC value was then calculated by averaging the values of three biological replicates. Data were presented for populations (whole culture) and individual colonies derived from respective populations.

### Biomass quantification

Production of biofilm biomass was quantified by the crystal violet assay (modified from [44]). Overnight cultures of the parental strain, evolved populations and individual colonies derived from these populations from the steady model were grown in BHI broth and diluted in 1/10 BHI broth to an absorbance of 0.05 AU (OD600_nm_). A 200 µl inoculum was transferred into a microtiter plate (Greiner, Kremsmünster, Austria). Microtiter plates were sealed with an adhesive gas-permeable membrane (Starlab, Hamburg, Germany) and incubated at 37 °C for 48 h, statically. Following the incubation period, the culture was removed, and the plates were washed with deionised water three times, air-dried, stained with 1% crystal violet solution (Sigma-Aldrich, St. Louis, MO, USA) and incubated for 15 min at room temperature. Next, 70% ethanol was added to the wells, and absorbance was determined for each well at OD590_nm_ using FLUOstar OMEGA plate reader (BMG Labtech, Ortenberg, Germany) in spiral averaging mode. Three biological and three technical replicates were included for each isolate. A sterile control well containing only the growth media was included in all experiments.

### Microscopy

Imaging was conducted exclusively on the *L. monocytogenes* parental strain as part of the optimization and validation of the bead-based biofilm persistence model. The purpose of this analysis was to confirm that reproducible biofilms could be established under the selected experimental conditions and to visualize their spatial organization on stainless-steel surfaces.

For both fluorescence and scanning electron microscopy, overnight cultures of the parental strain grown in BHI broth were diluted to an absorbance of 0.05 AU (OD600_nm_) in 1/10 BHI broth. Two millilitres of the diluted culture was added to each well of a 12-well polystyrene plate (Corning, NY, USA), each containing three stainless steel grade 316 coupons (10 mm diameter, 3 mm thick; LaserMaster, Redruth, Cornwall, UK). Coupons were incubated at 20 °C under mild agitation (60 r.p.m.) for 48 h to allow biofilm formation.

#### Fluorescence microscopy

Following incubation, coupons were gently washed with PBS to remove loosely attached cells and subsequently fixed for 30 min in 4% paraformaldehyde prepared in PBS. For staining, 100µl of a 5µM SYTO9 Green Fluorescent Nucleic Acid Stain (Thermo Fisher Scientific, Waltham, MA, USA) solution in PBS was applied to each coupon, followed by a 15-min incubation in the dark. Excess stain was removed by washing once with PBS. Coupons were then transferred into a µ-Dish (35 mm, high, glass-bottom dish; ibidi, Gräfelfing, Germany) equipped with an adhesive gene frame (25 µl, 1.0×1.0 cm; Thermo Fisher Scientific, Waltham, MA, USA).

Imaging was performed using a DeltaVision Elite Deconvolution Microscope (General Electric, Boston, MA, USA) equipped with a UPlanFLN Semi Apochromat 10× objective (Olympus, Hachioji, Tokyo, Japan), with a numerical aperture of 0.3. Fluorescence images were acquired using the FITC channel (excitation, 470/24 nm; emission, 525/36 nm) at a resolution of 512×512 pixels. Z-stack images were captured at 0.2-µm intervals. Four biological replicates were examined, with ten fields of view captured per replicate.

#### Scanning electron microscopy

Following incubation, coupons were gently washed with PBS to remove loosely attached cells and subsequently fixed in 2.5% glutaraldehyde diluted in 0.1M sodium cacodylate buffer for 1 h. A sterile coupon, prepared using the same procedure, served as a control to distinguish the coupon surface from biofilm biomass. After fixation, the fixative was removed, and coupons underwent three washes with 0.1M sodium cacodylate buffer. The coupons were then post-fixed in 1% osmium tetroxide for 1 h.

After three washes with distilled water, coupons were dehydrated through an ethanol dilution series (30%, 50%, 70%, 80%, 90% and 3 changes of 100% ethanol), each step lasting at least 15 min, with a final rinse and dry in 100% ethanol. Coupons were dried in an EM CPD300 Critical Point Dryer (Leica, Wetzlar**,** Germany) using liquid carbon dioxide as the transition fluid. Dried coupons were attached to aluminium SEM stubs using silver paint, coated with an 8.5 nm layer of gold in an ACE 600 sputter coater (Leica, Wetzlar**,** Germany), and immediately imaged in a Nova NanoSEM (FEI, Hillsboro, OR, USA) scanning electron microscope at 3 kV.

### DNA extraction

DNA was obtained from 1 ml of overnight cultures grown in BHI broth from populations (whole culture) and individual colonies. Prior to extraction, the cells were lysed in a custom lysis solution [20 mg ml^−1^ lysozyme (Roche, Basel, Switzerland)] and 1.2% Triton X-100 (Sigma-Aldrich, St. Louis, MO, USA) in H2O) and incubated at 37 °C for 1 h, followed by addition of 20 mg ml^−1^ RNase A solution (Promega, Madison, USA). DNA was extracted using the Maxwell^®^ RSC Cultured Cells DNA Kit (Promega, Madison, WI, USA) on the Maxwell^®^ RSC 48 automated extraction system according to the manufacturer’s instructions. DNA concentrations were measured using Qubit™ dsDNA Quantification Assay Kits (Thermo Fisher Scientific, Waltham, MA, USA).

### DNA sequencing

Sequencing was performed at the Quadram Institute (Norwich, UK). DNA was normalized to a concentration of 5 ng µl^−1^ and used to generate 150 bp paired-end sequencing libraries with the Illumina DNA Prep Kit (Illumina Inc., San Diego, CA, USA). Sequencing was performed on an Illumina NextSeq 500 instrument using Mid Output Flowcell and NSQ^®^ 500 Mid Output KT v2 (300 cycles). The short-read files are publicly available (BioProject: PRJNA1276474).

### Bioinformatic analysis

Paired-end short reads were filtered with trimmomatic (v0.38.0) (GitHub - usadellab/Trimmomatic) using default parameters with a sliding window of 4 bp and quality of 20 and inspected for contamination using kraken2 (v2.1.1) [45]. SNPs between the evolved lineages (entire population and individual colonies) and the parental strain were identified by comparing the trimmed paired-end reads and the annotated genome of BL87-028 (https://doi.org/10.5281/zenodo.14906828) in GenBank format using Snippy (v4.4.3) [46] using default parameters. Outputs were manually analysed in Microsoft Excel, and synonymous variants were excluded from further analysis. To identify SNPs unique to the biocide or biofilm conditions, we used an online Venn diagram tool [47] and selected only those SNPs that appeared exclusively in the conditions of interest. The genes carrying SNPs, their frequency and the number of SNPs per experimental condition were plotted using the RAWGraphs web tool [48]. SNP positions were labelled on the BL87-028 genome and visualized with SnapGene Viewer (v6.2.2).

### Conservation of nucleic acid residues

Conservation of the mutated residues within the *ykoK* leader was evaluated by comparison with homologous sequences from the National Center for Biotechnology Information (NCBI) database using blast [49]. Homologous sequences were retrieved (Table S1; Supplementary Material 2), excluding *L. monocytogenes* (taxid: 1639), to assess conservation within the *Listeria* genus. To assess conservation outside of the *Listeria* genus, sequences of related non-*Listeria* species were retrieved (Table S2). Multiple sequence alignment was performed in clustalw (v2.1) [50] on 50 sequences. Graphical representation of nucleic acid sequence alignment was generated with WebLogo (v.2.8.2) [51,52].

The alignment of the *ykoK* leader sequence against the *ykoK* leader from *Bacillus subtilis* IA40 (PDB: 2QBZ) was conducted using the Sequence Alignment Tool available on VectorBuilder (vectorbuilder.com). InterPro was used with default parameters to predict functional domains in analysed proteins against signatures from member databases [53]. Predicted protein structures were obtained from the AlphaFold Protein Structure Database [54,56], while experimentally determined structures were retrieved from the Protein Data Bank (PDB) [57]. Accession numbers were provided for all structures used in the analysis.

### Statistical analysis

All data were analysed using GraphPad Prism 5.4, and results are expressed as mean±sd, unless stated otherwise.

Statistical comparisons between multiple groups were performed using one-way ANOVA, followed by Tukey’s post hoc test for pairwise comparisons, when data passed the normality test with the Shapiro–Wilk test and had a variance ratio below 4. In cases where data did not pass suitability for ANOVA, it was tested with the Kruskal–Wallis test followed by Dunn’s multiple comparison post hoc test. Statistical significance was set at *P*<0.05, with significance levels indicated as follows: *P*<0.05 (*), *P<*0.01 (**), *P*<0.001 (***) and *P*<0.0001 (****).

## Results

### Establishment of a biofilm persistence model for *L. monocytogenes*

The model involves biofilm formation on a solid substrate (bead) suspended in liquid culture media, followed by biofilm detachment and re-establishment through sequential bead passaging. This process is repeated under selective pressure (i.e. exposure to sub-inhibitory concentration of biocide) with the opportunity to characterize evolving populations of *L. monocytogenes* using whole-genome sequencing. To establish a robust and reproducible BPM, we optimized several parameters, including (1) incubation time for consistent biofilm formation, (2) sub-inhibitory concentration of BC, (3) long-term stability of bacterial populations in the presence of BC and (4) differentiation between the beads from alternating passages.

To determine the optimal incubation period for biofilm formation, we quantified the total population of *L. monocytogenes* parental strain supported on a 6-mm bead in 1/10 BHI broth. We selected a 48-h incubation period, which yielded 10^5^–10^6^ c.f.u. bead^−1^, a range previously described to be sufficient for stable biofilm carry-over between passages [42].

**Fig. 1.**
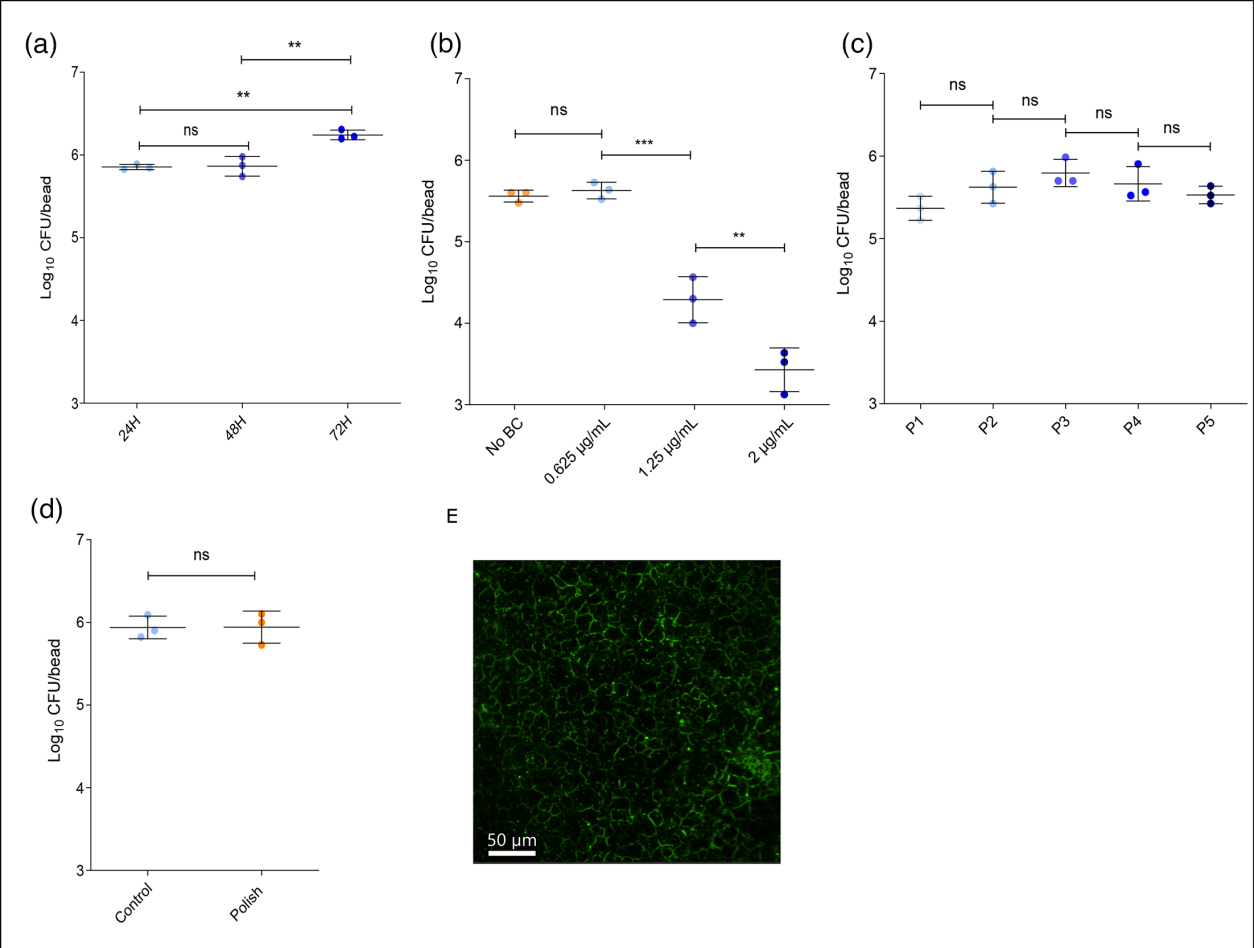
Optimization and validation of the biofilm persistence model. (**a**) Selection of sub-inhibitory BC concentration. Biofilm formation on stainless steel beads was assessed following 48-h incubation with 0, 0.625, 1.25 and 2 µg ml^−1^ BC. The sub-inhibitory concentration of 0.625 µg ml^−1^ maintained the population size of the parental strain within the desired range of 10⁵−10⁶ c.f.u. bead^−1^. (**b**) Model stability across passages. Using 0.625 µg ml^−1^ BC, the stability of the biofilm model was confirmed over five consecutive 48 h passages [passage 1 (P1) to passage 5 (P5)] by measuring c.f.u. bead^−1^ on stainless steel beads. The population remained stable in the presence of BC throughout all passages. (**c**) Validation of bead marking method. Beads were marked with nail polish to distinguish between the beads from alternating time points, and their potential impact on bacterial attachment was evaluated. The marking had no detectable effect on the ability of the parental strain to attach to the bead surface. (**d**) Fluorescence microscopy of the parental strain biofilm. Representative two-dimensional top-view fluorescence image of a 48-h biofilm formed by the parental strain in 1/10 BHI broth at 20 °C, stained with SYTO9, and visualized on stainless-steel (SS316) coupons at 10× magnification. Scale bar is 50 µm. All experiments were performed in triplicate. Dots represent mean values of three technical replicates. Horizontal bars indicate the mean of three biological replicates±sd. Statistical analyses were performed using one-way ANOVA followed by Tukey’s post hoc test (**P<0.01, ***P<0.001); ns, not significant.

We then identified a BC concentration that could support a population density on the beads comparable to non-exposed control. The mean MIC value for planktonic cells was determined to be 3.12 µg ml^−1^ of BC. We tested the effect of sub-inhibitory BC concentrations below the MIC level (2, 1.25 and 0.625 µg ml^−1^) on biofilm-forming capacity on beads. A concentration of 0.625 µg ml^−1^ BC was selected, as it did not significantly reduce the number of viable cells attaching to the bead surface compared to the non-exposed control (Fig. 1a). Bacterial populations were also maintained over the course of five passages with a 48-h incubation period between the passages, at 0.625 µg ml^−1^ BC, further validating that this is a sub-inhibitory concentration (Fig. 1b).

We also confirmed that the marking for differentiation between alternating beads from subsequent passages did not significantly impact bacterial attachment to the surface of stainless-steel beads (Fig. 1c).

To validate biofilm formation and spatial distribution under BPM conditions, we used fluorescence and scanning electron microscopy (SEM) during model optimization. Biofilms formed on stainless-steel coupons were stained with the nucleic acid dye SYTO9 and examined by fluorescence microscopy. The biofilms appeared thin and lacked pronounced three-dimensional structures. Notably, the biofilm displayed a distinctive honeycomb-like hexagonal arrangement (Fig. 1d), likely resulting from cells aligning along microcracks and crevices in the coupon surface. This interpretation was corroborated by SEM, which confirmed the topographical features of the coupon surface (Fig. S2).

### Exposure of *L. monocytogenes* to BC leads to the emergence of planktonic and biofilm lineages with reduced susceptibility

To investigate the adaptive response of *L. monocytogenes* to BC, we first examined how the steady model supported bacterial adaptation over time. The parental strain was exposed to a constant concentration of 0.625 µg ml^−1^ BC across eight independent lineages, with four biologically independent lineages evolving under either planktonic or biofilm conditions. Additionally, four non-exposed control lineages were included under both conditions. The steady model was run for 30 passages, with 3 key time points selected for BC susceptibility characterization: early (passage 11, P11), mid (passage 22, P22) and late (passage 30, P30). At each time point, BC tolerance was assessed in both *populations* (whole evolved cultures) and in *individual colonies* (randomly isolated colonies per lineage). Fold changes in mean MIC values were calculated relative to the parental strain (Fig. S1).

**Fig. 2.**
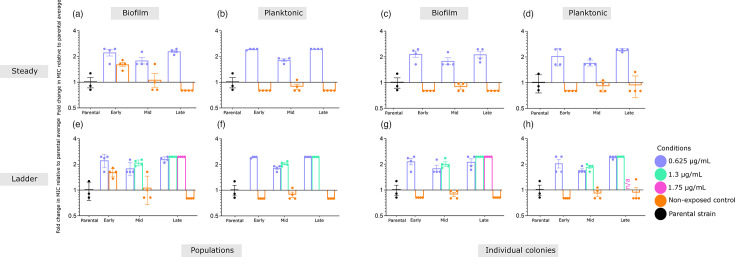
Exposure of *L. monocytogenes* to BC results in the emergence of tolerant planktonic and biofilm lineages. Fold change in average MICs of BC-exposed and non-exposed lineages, relative to the average MIC of the parental strain, across early (P11), mid (P22) and late (P30) evolutionary passages, under the steady (a–d) and ladder (e–h) models. Data are presented for both populations (a–b, e–f) and individual colonies (c–d, g–h). MIC values were measured in triplicate for each lineage. For populations, results from four independent lineages were averaged. For individual colony populations, the mean MIC from individual representative colonies is shown. Error bars represent standard error. n/a, not applicable.

Following repeated exposure to BC in the steady model, evolved lineages exhibited reduced susceptibility to BC (Fig. 2a–d). In all BC-exposed populations derived from both populations and individual colonies, mean MIC values were significantly higher than those of the non-exposed controls and the parental strain, except early biofilm populations (Fig. 2a). When analysing dynamics in reduced susceptibility across evolutionary time points, no significant change in MIC values was observed among populations (Fig. 2a–b). MIC values for non-exposed control lineages remained consistent across all time points and were comparable to the parental strain (Fig. 2a–d).

To further evaluate the resilience of evolved lineages under escalating BC stress, we employed the ladder model. Lineages initially exposed to 0.625 µg ml^−1^ BC during the first 11 passages were subjected to an increase to 1.3 µg ml^−1^ BC and further evolved until the late passages (P30) (1-step ladder model). All BC-exposed populations showed higher MIC values than non-exposed controls. However, no additional MIC increase was detected in either biofilm (Fig. 2e, g) or planktonic (Fig. 2f, h) populations at mid (P22) or late (P30) passages compared to those exposed to 0.625 µg ml^−1^ BC.

Lineages exposed to 1.3 µg ml^−1^ BC between early (P11) and mid (P22) passages underwent a second concentration increase to 1.75 µg ml^−1^ BC from mid (P22) to late (P30) passages (2-step ladder model). Under these conditions, only biofilm lineages were recovered, while planktonic lineages could not be maintained. Biofilm lineages exposed to 1.75 µg ml^−1^ BC maintained MIC values comparable to those previously exposed to 1.3 µg ml^−1^ (Fig. 2e, g), but higher than the average MIC of the non-exposed control.

### Genomic adaptations in *fepR* and 5′ UTR associated with reduced BC susceptibility across planktonic and biofilm conditions

**Fig. 3.**
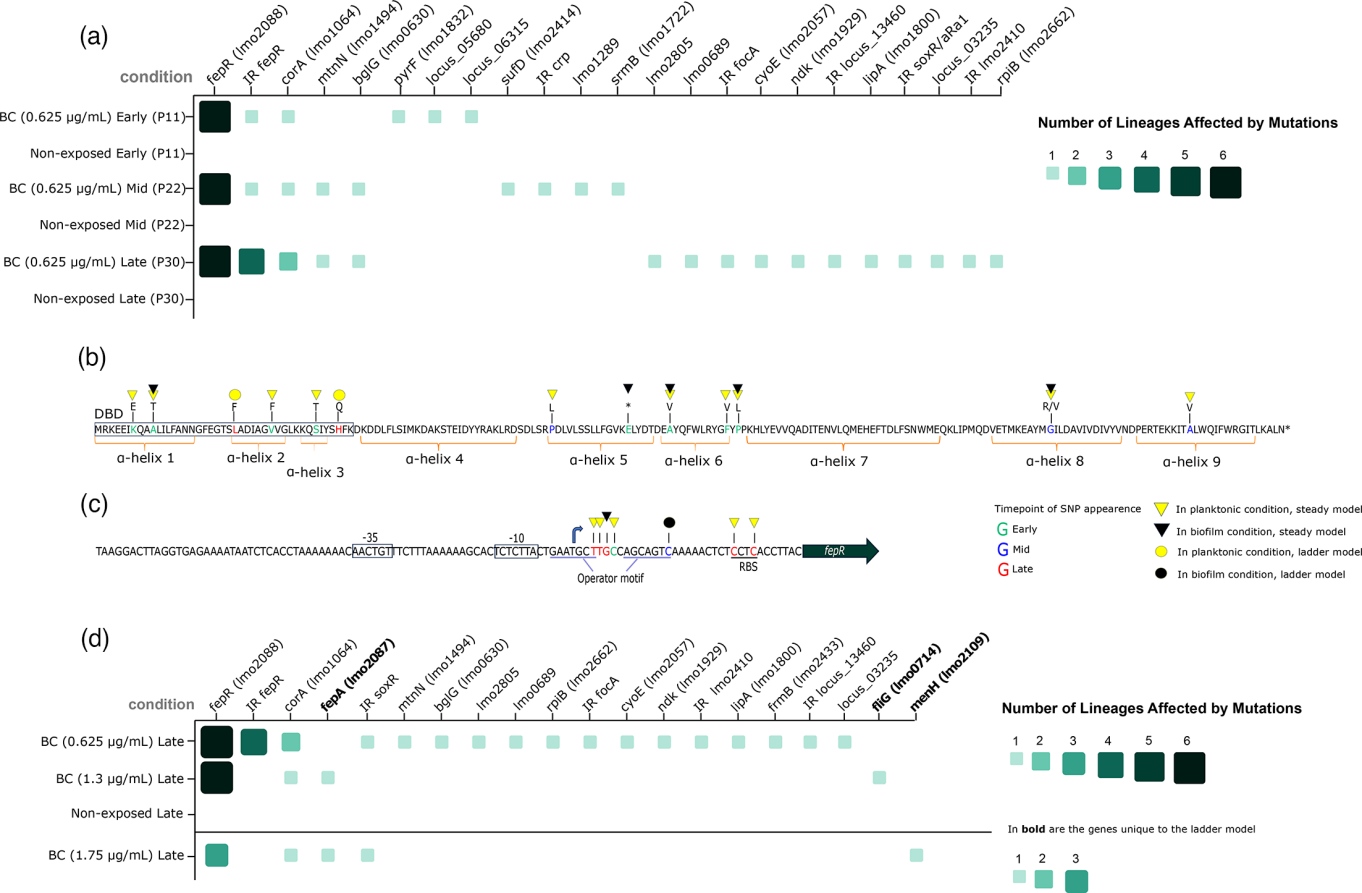
Mutations in the *fepRA* operon promote reduced susceptibility to BC in *L. monocytogenes* under steady and escalating exposure. (**a**) Mutation frequency scheme for SNPs acquired under steady exposure to BC (0.625 µg ml^−1^) (conditions ‘BC’) compared to non-exposed control (condition ‘No BC’) at different evolutionary time points: early (passage 11), mid (passage 22) and late (passage 30). Data encompasses six biologically independent lineages for BC-exposed and six for BC-non-exposed conditions at each time point. (**b**) Distribution and predicted domain location of non-synonymous mutations within *fepR* under steady and ladder BC exposure models. Gained residues are indicated above the sequence (amino acid map). Amino acids are colour-coded according to the phase in which the mutations were first detected: green, early phase; blue, mid phase; red, late phase. * indicates gain of an early stop codon. A yellow triangle represents mutations that occurred in planktonic lineages. Black triangle, in biofilm lineage; yellow and black circles, in the ladder model. N-terminal DNA binding domain (DBD) is indicated by a rectangle (amino acid residues 1–42). C-terminal effector binding domain spanning *α*-helices 4 to 9. Structural elements are highlighted below the sequence. (**c**) Map of base pair substitutions within 5′ UTR of *fepR* occurring in steady and ladder BC exposure models. Several functional elements were colour coded: blue arrow indicates the transcription start site (TSS), promoter regions −35 and −10 are indicated by a rectangle, the putative FepR operator motif is underscored in light blue, and the ribosome-binding site (RBS) is underscored in black. (**d**) Mutation frequency scheme for SNPs acquired under increasing exposure to BC (ladder model, 0.625–1.3to 1.75 µg ml^−1^) at late evolutionary time point. Biologically independent lineages=6 (0.625–1.3 µg ml^−1^) and 3 (1.75 µg ml^−1^).

To investigate the genetic changes associated with the emergence of reduced susceptibility to BC, three evolved lineages from each time point (Table S3) under both planktonic and biofilm conditions underwent whole-genome sequencing (for each lineage, entire populations and three individual colonies were sequenced). This was performed for both BC-exposed and non-exposed control lineages. Non-synonymous mutations were identified by comparing short reads from evolved lineages to the annotated parental strain (https://doi.org/10.5281/zenodo.14906828). Mutations occurring in BC-exposed lineages from both biofilm and planktonic conditions across three time points are illustrated in Fig. S3. The Venn diagram highlights the overlap and condition-specific distribution of genes carrying SNPs, distinguishing shared adaptive targets from those unique to either biofilm or planktonic lifestyles. Additionally, to visualize the frequency of SNPs, mutations were merged, encompassing six independent lineages, which were compared to mutations in non-exposed control lineages (Fig. 3a) (to view non-synonymous mutations that exclusively were detected in non-exposed lineages, see Fig. S5). Regarding non-synonymous mutations detected in non-exposed lineages, these mutations did not appear in any of the BC exposed conditions and, therefore, did not interfere with the interpretation of the results on adaptation to BC exposure. Notably, mutations in the intergenic region of *celB*, involved in metabolism of complex carbohydrates [58], were detected in both biofilm and planktonic non-exposed lineages and were not observed in any other condition, which may indicate adaptation specifically to a disinfectant-free environment.

In the steady model, all six BC-exposed lineages (biofilm and planktonic) independently acquired mutations in *fepR* (*lmo2088*), a local repressor of the MATE fluoroquinolone efflux pump FepA, whereas no mutations in *fepR* were detected in non-exposed lineages. These mutations rapidly reached fixation in all exposed populations and remained stable across subsequent passages (Fig. 3a). When analysed separately (Fig. S4), *fepR* mutations were found to occur with similar frequency in both biofilm- and planktonic-derived populations.

Overall, we detected 12 non-synonymous mutations (at 11 positions) within *fepR* in response to a steady concentration of BC (14 non-synonymous mutations at 13 positions in total for steady and ladder models) (Fig. 3b). We did not identify more than one mutation within *fepR* in the same isolate. Planktonic lineages exhibited a greater diversity of mutations (11 mutations) compared to biofilm lineages (5 mutations). Seven mutations appeared during early passages (K7E, A10T, V30F, S37T, A96V, F105V and P107L), three emerged at mid passages (L76P, G157R and A181V), with G157R also detectable until the final time point (Fig. 3b). Four mutations were shared between biofilm and planktonic lineages, three in early passages (A10T, A96V and P107L) and one in mid passages (G157V). Notably, a single mutation (E89*), introducing a premature stop codon, was exclusively detected in one biofilm lineage. Among the 12 identified mutations, five (K7E, A10T, L24F, V30F and S37T) were located within the N-terminal DNA-binding domain (DBD) (residues 1–42) [59], including two (V30F, S37T) within the HTH motif (residues 23–42). In particular, the A10T substitution from a non-hydrogen-forming methyl to a hydrogen-forming hydroxyl group leads to a gain of a hydrogen bond-forming ability by the side chain (Table S4).

The remaining seven mutations occurred in the C-terminal effector-binding domain, spanning *α*-helices 4 to 9 (Fig. 3b). Mutations at position 157 (G157R/V) resulted in two independent amino acid substitutions.

Beyond coding regions, seven mutations were identified in the *fepR* 5′ UTR (Fig. 3c). A single mutation arose in a planktonic lineage during early passages within the intra-operator spacer, while another was detected in a biofilm lineage at mid passages within the operator. Five mutations (four in planktonic lineages and one in a biofilm lineage) appeared in late passages, distributed across the putative ribosome-binding site, the intra-operator spacer and the operator (Fig. 3c). Two mutations located in the left side of the putative operator motif were predicted to overlap the transcription start site (TSS) occurring at the third and 14th base pairs downstream of the TSS.

### Mutations affecting metal homeostasis (*corA*) and metabolic pathways (*bglG*; *mtnN*) were detected in BC-exposed and biofilm-associated populations

The *corA* gene (*lmo1064*), encoding a metal cation transporter [60], emerged as another candidate for reduced susceptibility to BC exclusively in biofilm lineages. A total of four distinct non-synonymous mutations were detected in two independent BC-exposed biofilm lineages, with one mutation (S166P) arising in early passages, two (T127K and L292F) in mid passages and one (P268Q) in late passages. Notably, mutation L292F was predicted to reside within a membrane-embedded region (InterPro) (Figs 3a, S6A).

A mutation within the *mtnN* gene (*lmo1494*), encoding 5′-methylthioadenosine nucleosidase (UniProt: Q9R4A1), was observed at position 180 (A180T) in a single biofilm lineage during mid and late evolutionary passages (Figs 3a, S6B). Similarly, mutations in the *bglG* gene (*lmo0630*), encoding a transcriptional regulator of the *bgl* operon (phosphotransferase system) [61], were detected in a single biofilm lineage at mid and late passages at position 29 (E29V) (Figs 3a, S6C).

Additional mutations were identified exclusively in BC-exposed lineages, though these only appeared at a single time point. In early passages, such mutations were found in *pyrF* (*lmo1832*), *lmo1040* and *lmo1183*. In mid passages, mutations occurred in *sufD (lmo2414)*, *srmB (lmo1722)*, *lmo1289* and the intergenic region upstream of *crp*. In late passages, mutations were detected in *lmo2805*, *lmo0689*, *rpiB (lmo2662)*, *cyoE (lmo2057)*, *ndk (lmo1929)*, *lipA (lmo1800)* and *fliG (lmo0714)*, as well as in the intergenic regions upstream of *lmo2410*, *soxR (lmo2722)* and *focA (lmo0593)* (Fig. 3a).

### Stepwise BC escalation does not affect the level of reduced susceptibility to BC but selects for *fepA* mutations, potentially counteracting *fepR* effects

We further examined genetic traits associated with adaptation under progressively increasing BC concentration in the ladder model. As with the steady model, mutations occurring in BC-exposed lineages from both biofilm and planktonic conditions were grouped for these analyses, encompassing six independent lineages. Mutations identified in early and late passages from the steady model served as a reference for comparison with the ladder model. Overall, mutations were detected in six genes in lineages exposed to escalating BC concentrations, including *fepA*, *fliG* and *menH*, which were not affected in the steady model.

Mutations in lineages exposed to the 1-step ladder model (0.625 μg ml^−1^ to 1.3 μg ml^−1^) were described only for the late passages. All six lineages continued to exhibit mutations in *fepR* (Fig. 3b, d), including two additional SNPs (L24F and H41Q), which were not identified in the steady model (Fig. 3b). Notably, both mutations were located within the HTH motif of the DBD (spanning amino acid residues 23 to 42). In contrast, three SNPs found in *fepR* in lineages evolving at 0.625 µg ml^−1^ (A96V, P107L and G157R/V) were not detected in lineages exposed to 1.3 µg ml^−1^. Similarly, a mutation in the *fepR* 5′UTR intra-operator spacer, observed in one lineage exposed to 0.625 µg ml^−1^ BC, was lost following exposure to 1.3 µg ml^−1^ BC (Fig. 3c).

Such BC escalation was selected for two new SNPs (H439FS and W70L) in one biofilm lineage within *fepA (lmo2087)*, a MATE efflux pump gene downstream of *fepR* (Fig. 3d). The H439FS substitution resulted in a frameshift, while the W70L mutation was located on an α-helix within a membrane-embedded region of FepA (spanning amino acid residues 49 to 73) (InterPro) (Fig. S6D). In both cases, mutations in *fepA* co-occurred with a gain of an early stop codon in *fepR* (E89*), suggesting a potential compensatory relationship between the two genes.

Additional mutations in several genes were exclusive to the ladder model. In *corA*, a S166P mutation observed in a biofilm lineage prior to BC escalation was no longer present after the increase in BC concentration. However, a new W290C substitution emerged in a biofilm lineage when the concentration increased to 1.3 µg ml^−1^ BC. Notably, mutation W290C was predicted to reside within a membrane-embedded region (InterPro) (Figs 3d, S6A).

For lineages exposed to a second BC concentration escalation (1.3 to 1.75 μg ml^−1^), mutations were described at late passages in three biofilm lineages, as growth was inhibited in planktonic lineages exposed to 1.75 µg ml^−1^ BC.

In *menH (lmo2109)*, encoding 2-succinyl-6-hydroxy-2,4-cyclohexadiene-1-carboxylate (SHCHC) synthase [62] acquired a T44I substitution (Fig. S6E). While in *fliG (lmo0714)*, a gene involved in flagellar motor function (UniProt: P0ABZ1), a frameshift was acquired (M188FS) (Figs 3d, S6F).

The *corA* W290C and *menH* T44I substitutions, previously observed in late passages at 1.3 µg ml^−1^ of BC, were also observed in populations exposed to 1.75 µg ml^−1^ of BC (Fig. S6A, E), suggesting they emerged prior to the second escalation.

### Mutations in genes related to metal homeostasis, pyrimidine synthesis and stress response underline biofilm persistence

To investigate genetic traits associated with biofilm persistence, the steady model dataset was reanalysed by grouping mutations based on lifestyle rather than BC exposure. Mutations identified in six independent biofilm lineages (three BC-exposed and three non-exposed) were compared to those detected in six independent planktonic lineages (three BC-exposed and three non-exposed) across every evolutionary time point (Table S5).

**Fig. 4.**
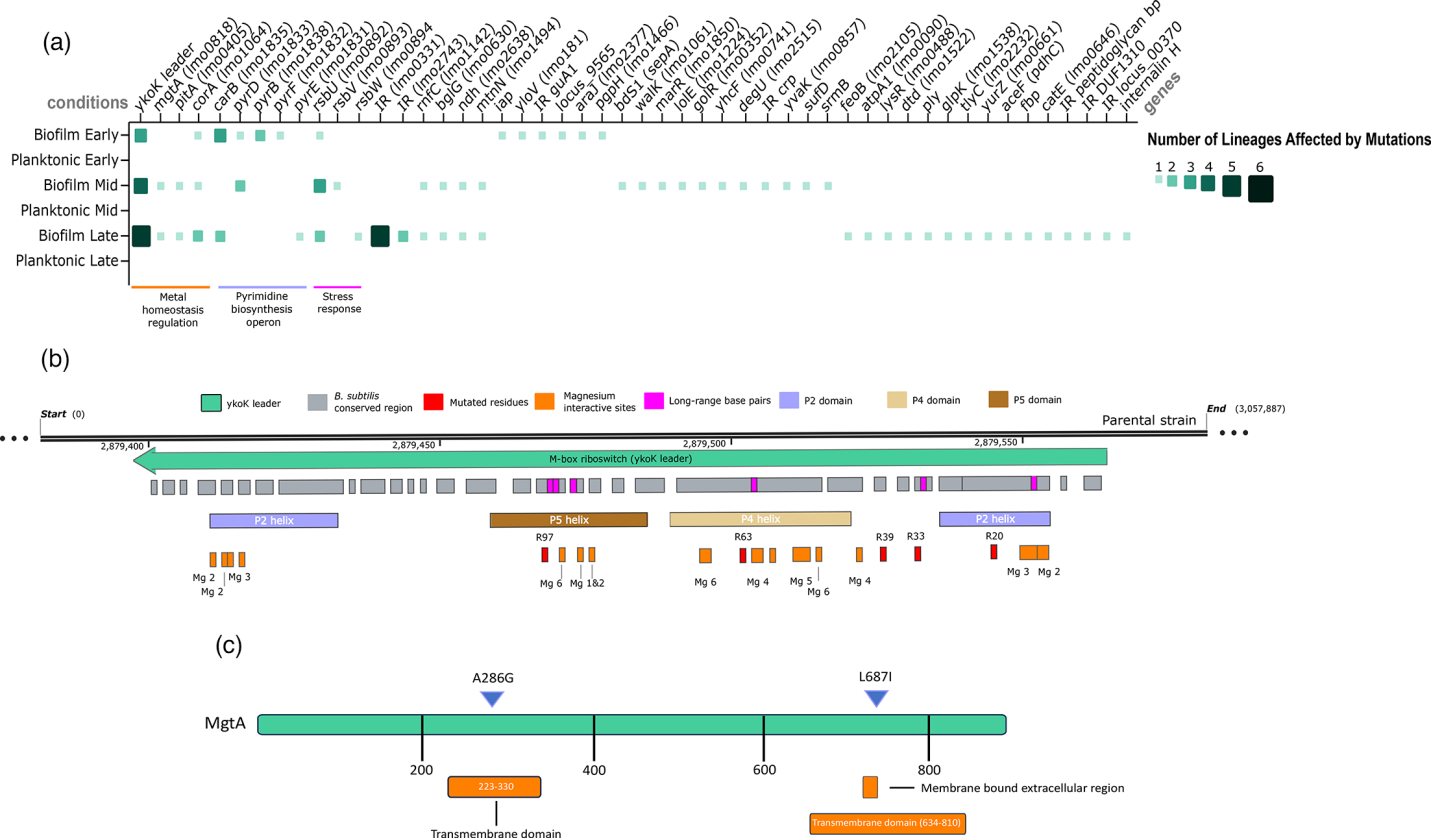
Mutations associated with metal homeostasis, pyrimidine synthesis and stress response in biofilm lineages. (**a**) Mutation frequency heatmap for SNPs acquired exclusively in biofilm lineages. The number of independent lineages carrying mutations linked to biofilm persistence is compared to planktonic controls. Six biologically independent biofilm and planktonic lineages were analysed. Evolutionary time points are categorized as early, mid and late phases. Colour intensity and square size indicate the number of independent lineages containing a SNP at each locus, with darker/larger squares representing higher frequency. Absence of squares indicates no detected mutations. (**b**) Structural mapping of the YkoK riboswitch leader and mutated residues. Conserved nucleotides between the parental strain and *B. subtilis* are shown in grey. Structurally important domains and putative magnesium-binding sites, inferred from *B. subtilis*, are indicated. YkoK predicted structure was obtained from NCBI: 2QBZ and previous studies [63]. (**c**) Overview of amino acid substitutions detected in MgtA in biofilm-associated lineages across early, mid and late time points. Substitutions are indicated by triangles, with predicted structural and functional domains annotated based on InterPro predictions.

Among the genetic elements associated with biofilm persistence, dispersion, growth and related to metal ion homeostasis were *ykoK* leader, *mgtA (lmo0818)*, *pitA (lmo0405)* and *corA. ykoK*, which encodes the M-Box riboswitch RNA, exhibited five mutations at nucleotide residues 20, 33, 39, 63 and 97 exclusively in biofilm lineages (Fig. 4a–b). These mutations accumulated over time. Three mutations were observed during early passages in three lineages, one additional mutation emerged in a fourth lineage during mid passages and a further mutation was detected in a fifth lineage during the late passages. Analysis based on the complete structure of the *ykoK* leader from *B. subtilis* IA40 (PDB: 2QBZ) revealed that all five mutations mapped to regions conserved between *B. subtilis* IA40 and the *L. monocytogenes* parental strain (Fig. 4b). Notably, nucleotide residues 20, 63 and 97 are positioned near Mg^2+^-interacting sites that are critical for maintaining the RNA’s structural conformation [63], while residues 33, 63 and 97 are also located near long-range base pairs that stabilize the termination loop. Comparative analysis of *ykoK* sequences across related species (Table S1 and S2, Fig. S7) showed that in Gram-positive species outside the *Listeria* genus, residues 20 and 63 are fully conserved, while residues 33, 39 and 97 are more variable (Fig. S7B). Within the *Listeria* genus, residues 20 and 97 are highly conserved, while substitutions are tolerated at positions 33, 39 and 63 (Fig. S7A). The high conservation of residue 20 across both *Listeria* and other Gram-positive species suggests that mutations at this position are likely to have significant functional impacts.

Two non-synonymous mutations (A286G and L687I) were detected in *mgtA*, in a single biofilm lineage (Fig. 4a). *mgtA* encodes a divalent metal transporter [64] located downstream of *ykoK*. Mutations in A286G (emerged during mid passage) and L687I (emerged during late passage) were both predicted to reside within transmembrane domains (residues 223–330 and 634–810, respectively) (Fig. 4c). L687I substitution co-occurred with mutations in *ykoK* at residue 37*,* while A286G substitution was detected independently*.* Notably, the L687I substitution from a hydrophilic, positively charged amino group to a hydrophobic, neutral isopropyl group leads to a loss of a hydrogen bond-forming ability by the side chain (Table S6). Non-synonymous mutations were also detected in *pitA*, encoding a low-affinity inorganic phosphate transporter [65,66]. The I58M substitution was detected in a single biofilm lineage during mid and late passages (Fig.re S8A) and notably co-occurred with a mutation in *ykoK* at residue 33.

**Fig. 5.**
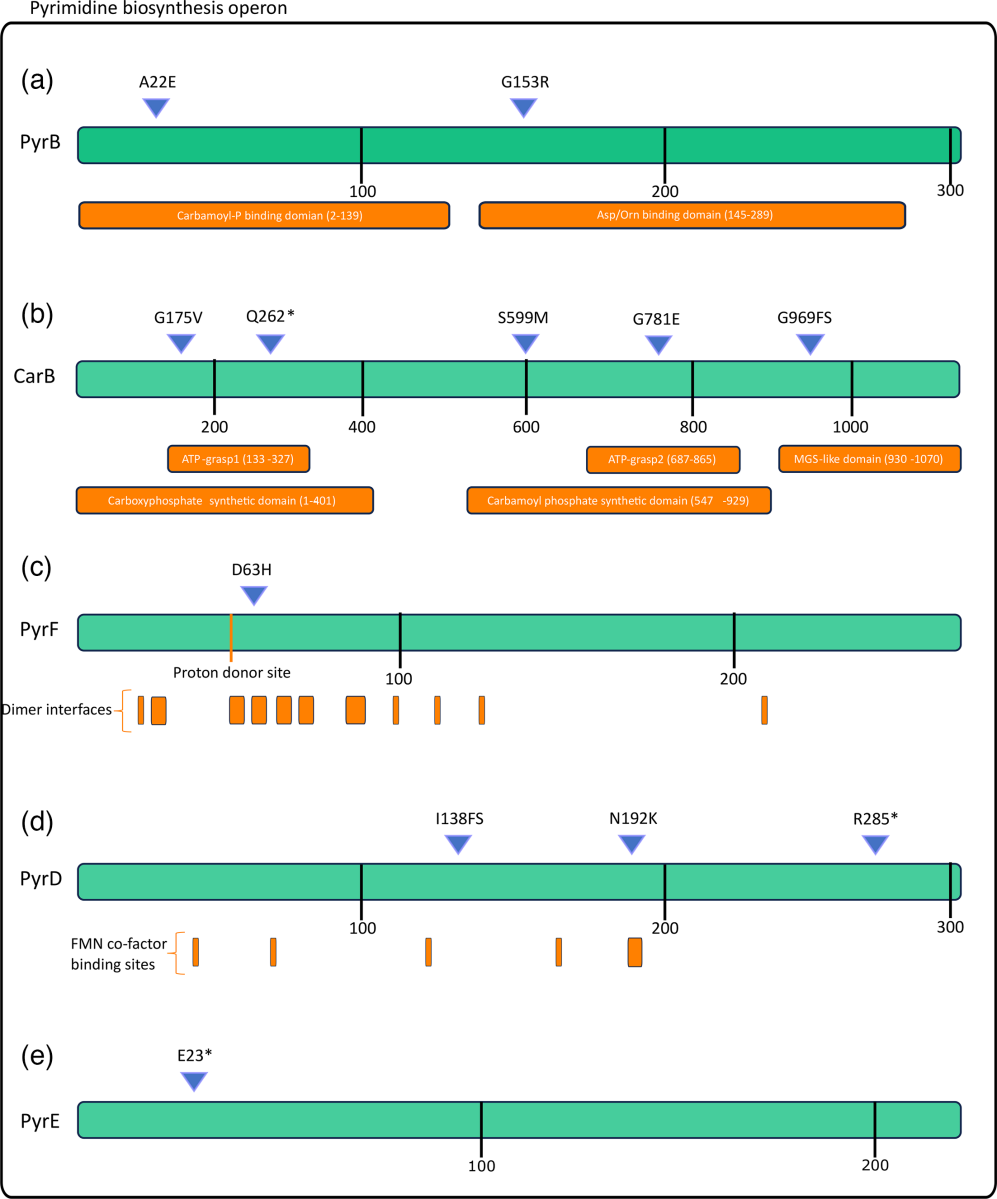
Amino acid substitutions in pyrimidine synthesis operon genes detected exclusively in biofilm lineages of the biofilm persistence model experiment. Graphical overview of substitutions detected in early, mid and late time points during biofilm adaptation. Structural and positional mapping of mutations is shown for (**a**) PyrB, (**b**) CarB, (**c**) PyrF, (**d**) PyrD and (**e**) PyrE. Mutations are indicated by triangles, with orange boxes highlighting affected regions. * indicates an early stop codon, and FS denotes a frameshift mutation. Predicted structural and functional domains were annotated using InterPro.

Non-synonymous mutations were also identified in five genes of the pyrimidine biosynthesis operon: *pyrB (lmo1838)*, *carB (lmo1835)*, *pyrF (lmo1832)*, *pyrD (lmo1833)* and *pyrE (lmo1831)* (Fig. 4a and Fig. 5)

Mutations also accumulated in the SigB regulatory operon (*rsbU*, *rsbV* and *rsbW*) (Figs 4a, S9). *rsbU* (*lmo0892*) exhibited six unique non-synonymous mutations: one early (E94FS), three mid (E22G, T204M and L60FS) and two late (Y9H and S41*). Frameshifts (L60FS and E94FS) and the stop codon S41* likely cause loss of function. T204M, within the PPM domain, may disrupt metal coordination, while Y9H and E22G near the active site may destabilize substrate binding. In *rsbV* (*lmo0893*), a single S58R substitution occurs at the regulatory phosphorylation site, potentially affecting sigma-B activation [67]. In *rsbW*, a stop codon (Q22*) was detected in the same lineage as the *rsbU* mutation S41*, suggesting coordinated disruption of SigB regulation.

Eight mutations were identified in the non-coding region upstream of a gene homologous to *lmo0331* (39% identity with InlJ; PDB: 3BZ5) during late passages (Fig. 4a), potentially affecting gene expression. Additional mutations were found in *rnfC* (*lmo1142*), which encodes an oxidoreductase complex subunit involved in ion transport (UniProt: Q7VNT4) and *ndh* (*lmo2638*), encoding NADH dehydrogenase. A frameshift mutation in *rnfC* (V410FS) appeared in a single biofilm lineage during mid and late passages (Fig. S8B). Similarly, *ndh* mutations (A274T; A259P) were observed in the same lineage over the same time points.

Finally, additional mutations exclusive to biofilm-associated lineages were detected at single time points (Fig. 4a). Regions affected during the early passages included *iap*, *yloV (lmo1814)*, *araJ (lmo2377)*, *pgpH* (*lmo1466*), locus_9565 and the intergenic region upstream of *guA1*. In mid passages, mutations occurred in *bdS1* (*sepA*), *walk (lmo1061)*, *marR (lmo1850)*, *lolE (lmo1224)*, *golR (lmo0352)*, *yhcF (lmo0741)*, *degU (lmo0352)*, *yvaK (lmo0857)*, *sufD* and *srmB*. In late passages, mutations were detected in *feoB (lmo2105)*, *atpA1 (lmo0090)*, *lysR (lmo0488)*, *dtd (lmo1522)*, locus_12680 (l-alanoyl-d-glutamate peptidase), *glpK (lmo1538)*, *tlyC (lmo2232)*, *yurZ (lmo0661)*, *aceF (pdhC)*, *fbp*, *catE (lmo0646)*, *inlH* and the intergenic regions upstream of *lmo2743*, peptidoglycan bound protein (locus_15625), DUF1310 and locus_00370*.*

### Biofilm persistence did not elicit an increase in biomass

Evaluation of late passages of biofilm lineages revealed that neither cell attachment to the bead (Fig. S10A) nor overall biomass production (Fig. S10B) increased between lineages exposed to BC and those evolved in the absence of BC.

## Discussion

The purpose of this study was to reveal mechanisms of FPE persistence by developing an adaptive biofilm model that resembles the conditions in FPE and allows for the *in vitro* evolution of genetic traits in *L. monocytogenes*. The model simulated the application of biocides typical of cleaning and disinfection programmes, along with FPE conditions such as low nutrient availability, reduced temperature and surface characteristics. The study specifically focused on selective outcomes associated with reduced BC susceptibility and biofilm persistence. The decision to summarize overall mutational trends rather than track individual lineages reflects the strength of the dataset, which captures population-level convergence under repeated selective pressures.

Through our steady model, we determined that adaptation to sub-inhibitory BC concentrations consistently targeted FepR, in both planktonic and biofilm lineages. Consistent with our findings, Guerin et al. [33] and Bolten et al. [68] reported that single non-synonymous mutations in *fepR* led to a twofold increase in BC MIC compared to the parental strain, in addition to other short-term evolution studies in *L. monocytogenes*, which also reported the emergence of BC-tolerant mutants [69,70]. These previous studies primarily focused on planktonic populations and did not assess biofilm-specific or long-term adaptive processes. In contrast, our model captured a more integrated spectrum of adaptation, from early mutational events associated with reduced susceptibility to BC, to longer-term compensatory mechanisms, particularly within biofilm-associated populations.

We also detected multiple mutations within the 5′ UTR of the *fepRA* operon, including five within the operator motif and two in the ribosome-binding site. These findings align with recent work by Schulz et al. [71], which implicated the 5′ UTR in *fepA* derepression.

Using both biofilm and planktonic models allowed for a direct comparison between the two systems, with the main mechanism involving *fepR* detected in both. Notably, a higher number of mutated loci under BC exposure was identified in the biofilm model (16 loci, including *corA*, *mtnN* and *bglG*) compared to the planktonic model (eight loci). This difference highlights distinct adaptive patterns between the two growth modes and suggests that the biofilm model may offer advantages for similar experimental evolution studies. In addition to being more representative of real-world conditions, the biofilm model also enabled the detection of adaptive pathways that were not observed in the planktonic counterpart. Notably, the extended design of the ladder model enabled us to capture not only the early adaptive responses in *fepR*, but also potential compensatory changes in *fepA* that emerged in the later passages. These previously unreported mutations in *fepA* co-occurred with a premature stop codon mutation in *fepR* (E89*), observed only in late passages. We hypothesize that such mutations may reduce or disrupt FepA function, thereby counteracting the constitutive overexpression driven by inactivation of *fepR*. Given that *fepA* expression can increase by 6- to 64-fold depending on the growth phase, sustained overexpression may impose a significant metabolic burden associated with continuous efflux activity. Thus, a partial loss of function in *fepA* may confer a fitness advantage by fine-tuning reduced susceptibility while mitigating the fitness cost of efflux hyperactivity.

Upregulation of phosphotransferase system genes, including *bglG*, has been observed by others in BC-tolerant mutants [72]. We also observed a *bglG* mutation in a BC-exposed condition, hinting at a broader metabolic adaptation, which may lead to a slower metabolic rate and, as a result, reduce active antimicrobial targets. Notably, as *bglG* mutations were also detected in biofilm conditions, they may impact the sessile lifestyle.

Recent studies suggest that CorA in *Mycobacterium smegmatis* and *Escherichia coli* may facilitate the extrusion of multiple structurally diverse drug classes, thereby reducing the susceptibility of host cells to these antimicrobials [73]. Fixation of *corA* mutations in BC-exposed lineages during our evolution experiment suggests that similar reduced susceptibility mechanisms may be at play in *L. monocytogenes*.

Beyond BC adaptation, our model revealed parallel mutations occurring in genes involved in metal ion homeostasis, pyrimidine biosynthesis and stress response, which may contribute to biofilm persistence. Among the most consistent findings were mutations impacting magnesium homeostasis, involving the *ykoK* leader, *mgtA* and *pitA*. The *ykoK* leader, which encodes the M-box riboswitch, was particularly enriched with mutations across all time points. Located upstream of *mgtA*, the *ykoK* leader functions as a magnesium-sensitive riboswitch that is suggested to regulate transcription of downstream *mgtA* depending on intracellular magnesium levels [74]. While the structure of M-Box in *L. monocytogenes* remains unresolved, a structural model from *B. subtilis* has identified magnesium-binding sites required for conformational control [63]. In our study, mutations were located near three predicted magnesium-interactive sites within conserved regions between *L. monocytogenes* and *B. subtilis,* as well as at predicted long-range base pair sites, suggesting potential disruption of riboswitch folding. Such disruption may affect M-box sensitivity to intracellular magnesium concentrations and, consequently, transcriptional regulation of *mgtA.*

Alongside *ykoK* mutations, we observed co-occurring mutations in *mgtA* and *pitA*, particularly in predicted transmembrane domains, suggesting functional fine-tuning of ion transport activity. *mgtA* encodes a magnesium-specific P-type ATPase capable of magnesium uptake [64], while *pitA* encodes a phosphate transporter which mediates the proton-driven uptake of soluble neutral metal phosphate (MeHP0_4_) complexes [65]. Since magnesium forms metal phosphate salts, thereby reducing its bioavailability, altered uptake of phosphates via *pitA* may indirectly modulate intracellular magnesium concentrations [66]. Such coordinated changes likely reflect an adaptive strategy to balance magnesium acquisition with energy cost, a balance especially important in biofilms, where ionic gradients, nutrient limitations and metabolic stress differ significantly from planktonic conditions [75].

Our findings align with prior research highlighting the role of magnesium in biofilm development. Chalke et al. [76] reported that exposure to 50 mM magnesium enhanced *L. monocytogenes* biofilm formation and influenced later stages of biofilm development. Similarly, Nowak et al. [77] found that inactivation of *mgtB*, another magnesium transporter, led to the formation of a unique biofilm with a sandwich-like structure, likely due to the stabilization of the extracellular polymeric matrix. These observations suggest that magnesium availability may influence biofilm physiology through multiple mechanisms, including its role as a cofactor, a signalling element or a modulator of ionic balance, rather than through effects on growth alone [78]. Although the effects of magnesium on biofilms vary across bacterial species, either promoting or inhibiting biofilm formation depending on concentrations and environmental context [79,84], our results support a central role of magnesium homeostasis in *L. monocytogenes* biofilm persistence.

Another key adaptation pathway in biofilm lineages was *de novo* pyrimidine biosynthesis. Several genes in this pathway (*carB*, *pyrD*, *pyrF*, *pyrB* and *pyrE*) acquired mutations exclusively in biofilm lineages. Previous studies in other bacterial species have linked pyrimidine biosynthesis genes to biofilm formation. For example, *pyrD* inactivation in *E. coli* reduced flagellar motility and biofilm formation by downregulating the expression of both type 1 fimbriae and curli subunit genes [85]. Similarly, in *Xanthomonas citri* subsp. *citri*, *carB* was required for swimming motility and biofilm formation [86]. Likewise, in *Pseudomonas aeruginosa*, *de novo* UMP biosynthesis was necessary for biofilm formation [87]*.* Although this link has not been previously demonstrated in *L. monocytogenes*, our findings suggest that disruption of pyrimidine biosynthesis may serve as a signal of severe nutrient stress, leading to the downregulation of biofilm-associated genes and potentially triggering biofilm dispersal or a transition to planktonic growth, paralleling mechanisms observed in *P. aeruginosa* [88].

We also identified recurrent mutations in *rsbU*, a positive regulator of the alternative sigma factor SigB, which controls ~300 genes involved in stress resistance and survival [89]. Mutations in *rsbU* occurred across all three evolutionary time points in several biofilm lineages, but were absent in BC-adapted lineages, contrary to the observations of Douarre *et al.* [90]. This result suggests that *rsbU* mutations may specifically benefit the sessile lifestyle. Supporting this hypothesis, the deletion of *rsbU* has been linked to increased extracellular matrix production and the number of cells in the biomass of *L. monocytogenes* [91]. Notably, in our study, a premature stop codon in *rsbW* co-occurred within the same lineage as the S41* premature stop codon in *rsbU*. This pattern may indicate a compensatory mechanism, where the *rsbW* loss-of-function mutation mitigates the altered stress response imposed by the loss of *rsbU* function.

Collectively, we demonstrated that *L. monocytogenes* biofilms sensitive to low concentrations of common FPE disinfectants can rapidly develop a tolerant phenotype. Our findings also provide new insights into biofilm persistence genetic mechanisms within FPEs and the potential targets exploited by *L. monocytogenes* to establish and maintain niches under otherwise unfavourable conditions. Importantly, the use of an evolutionary model enabled us to capture compensatory mechanisms that are not detectable through single time-point analyses or short-term stress exposures. Future investigations should focus on characterizing the mechanisms associated with these targets and their roles in biofilm formation and persistence.

## Supplementary material

10.1099/mgen.0.001611 Supplementary Material 1.

10.1099/mgen.0.001611 Supplementary Material 2.

## Abbreviations

BC: benzalkonium chloride
BHI: Brain Heart Infusion
BPM: biofilm persistence model
DBD: DNA-binding domain
FPE: food production environments
MATE: multidrug and toxic compound extrusion
MIC: minimum inhibitory concentration
NCBI: National Center for Biotechnology Information
PDB: Protein Data Bank
SEM: scanning electron microscopy
ST121: sequence type 121
TSS: transcription start site
