# Supplementary material for: Evolved populations of Listeria monocytogenes related to biofilm formation and biocide stress in the context of food production environment niches
Source: Microb Genom. 2026 Jan 21;12(1):001611. doi: 10.1099/mgen.0.001611 (PMC12824596; doi:10.1099/mgen.0.001611)

Supplementary Figure 1. Overview of the experimental design of the biofilm persistence model (BPM). The evolution experiment started with four biologically independent colonies under experimental (BC-exposed biofilm and planktonic) and control (non-exposed biofilm and planktonic) conditions, which were passaged 30 times. Beads and cultures were stored after 11 (early), 22 (mid) and 30 (late) passages. Evolved samples were analysed with a range of phenotypic tests (biocide sensitivity testing, cell attachment capacity, and biomass production). Evolved populations and individual colonies underwent whole-genome sequencing, followed by identification of genetic changes compared to the hybrid genome assembly of the parental strain using genome alignment tool Snippy.

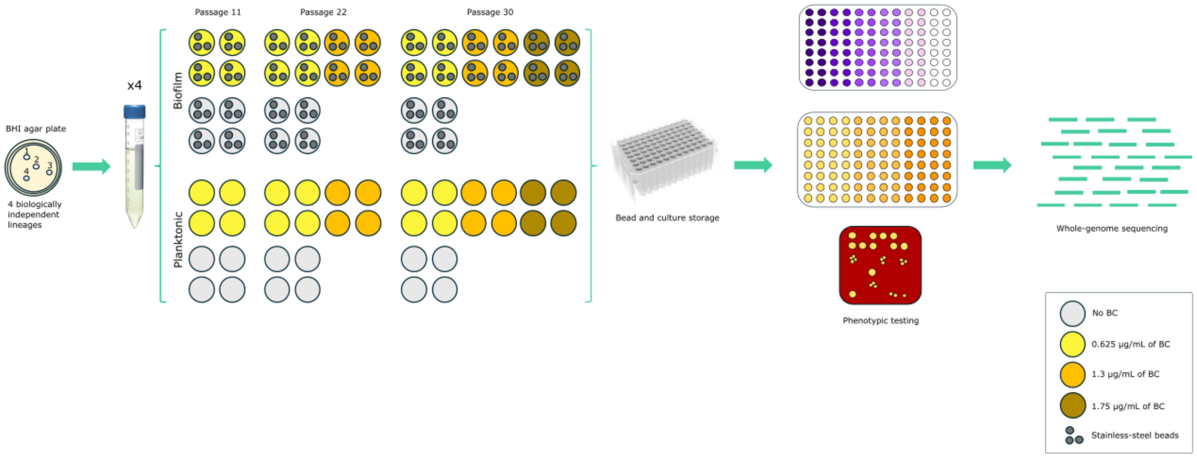

Supplementary Figure 2. Scanning electron microscopy (SEM) of stainless-steel surface topography. SEM image of a grade 316 stainless-steel coupon at 1000× magnification. The scale bar represents 50 µm. Surface features, including cracks and plateaus, are visible. The same surface was used for fluorescent microscopy imaging of the parental strain biofilm (Figure 1E).

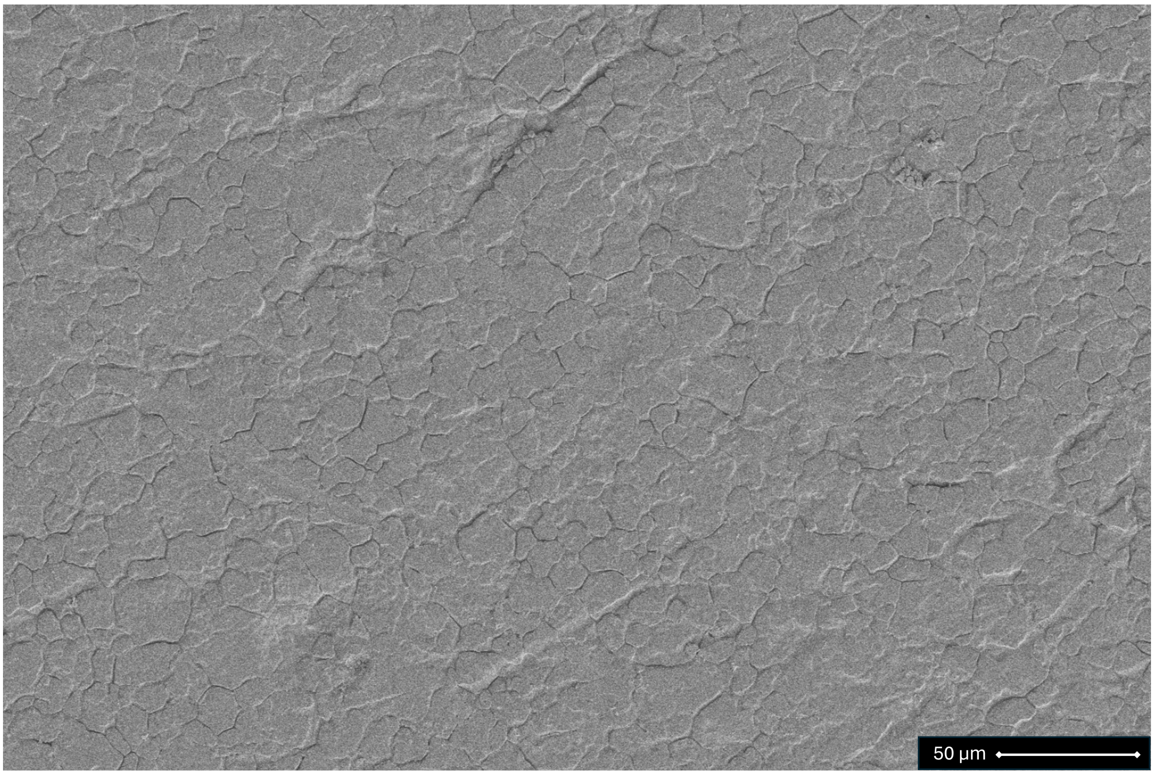

Supplementary Figure 3. Venn diagrams of loci and intergenic regions with mutations under continuous benzalkonium chloride exposure. Diagrams show genes and intergenic regions (IR, followed by the downstream gene name) in which SNPs were identified after continuous exposure to 0.625 µg/mL benzalkonium chloride (BC) in biofilm-associated and planktonically evolved lineages, as well as in unexposed controls. Mutations detected across **early**, **mid**, and **late** timepoints are included. Overlapping regions indicate mutations shared between conditions. Venn diagrams were generated using an online tool (Heberle et al., 2015).

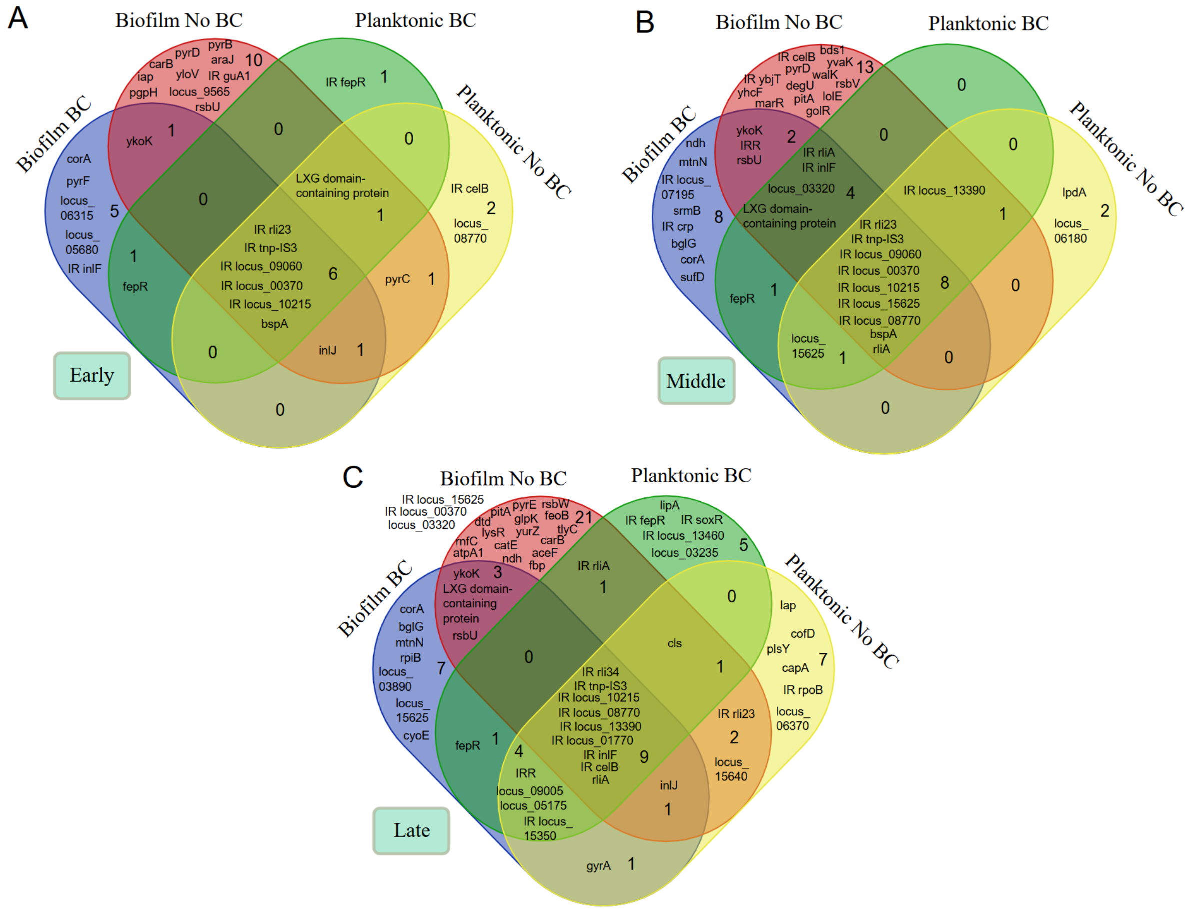

Supplementary Figure 4. Mutations in *fepRA* operon related to efflux activity become fixed in the BPM population under BC exposure, regardless of biofilm or planktonic evolution. Mutation frequency table demonstrating number of (**A**) **biofilm** and (**B**) **planktonic** lineages carrying genetic traits associated with prolonged exposure to BC (0.625 µg/mL) (conditions "BC") compared to non-exposed control (condition "No BC") at different evolutionary phases: early (Passage 11), mid (Passage 22) and late (Passage 30). Data for each condition represents 3 biologically independent lineages.

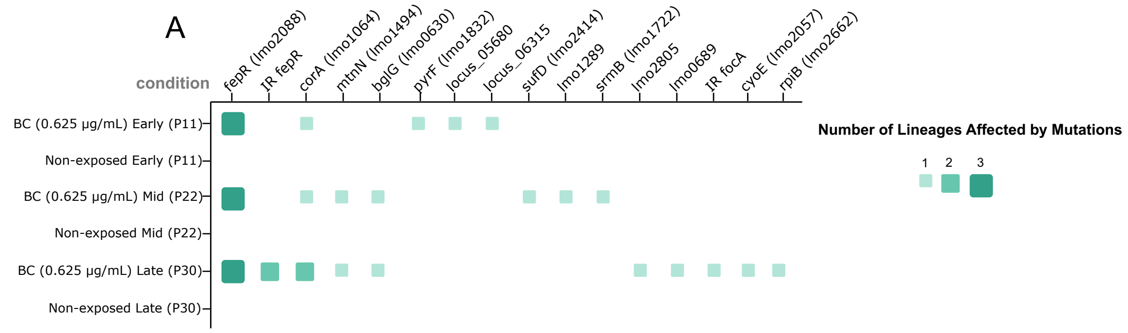

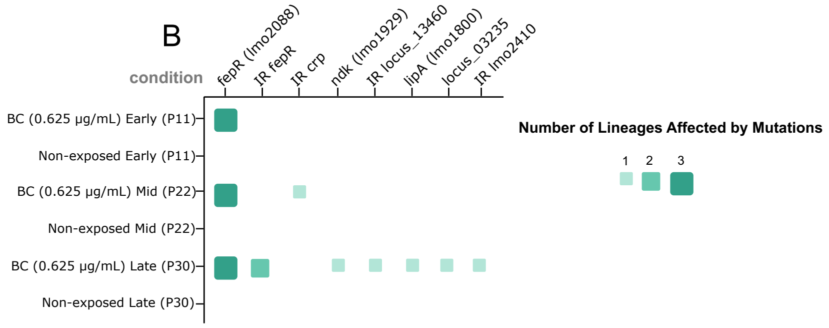

Supplementary Figure 5. Mutations that were detected exclusively in non-exposed biofilm and planktonic lineages of the biofilm persistence model. Mutation frequency table demonstrating number of (**A**) **biofilm** and (**B**) **planktonic** lineages carrying genetic traits associated with no exposure to BC (conditions "non-exposed") compared to BC exposed group (condition "no BC") at different evolutionary timepoints: early (Passage 11), mid (Passage 22) and late (Passage 30). Data for each condition represents 6 biologically independent lineages.

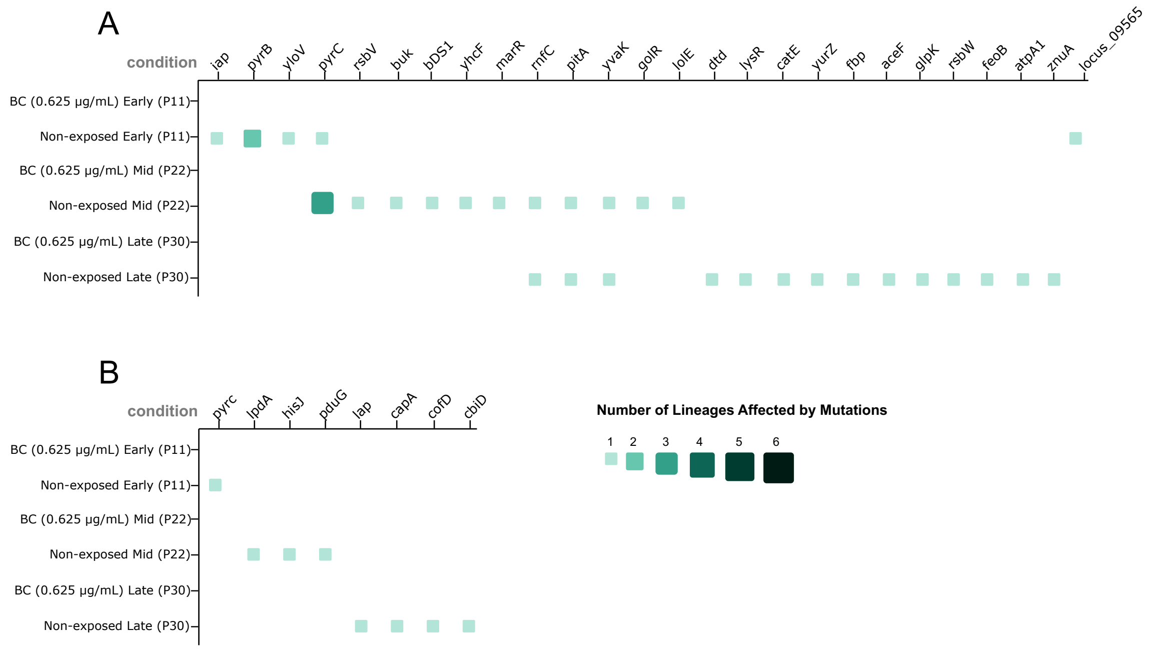

Supplementary Figure 6. Graphical overview of amino acid substitutions in (A) CorA, (B) MtnN, (C) BglG, (D) FepA, (E) MenH, (F) FliG. Non-synonymous mutations are indicated by triangle. Affected domains are demonstrated in orange boxes. Domains were predicted with InterPro.

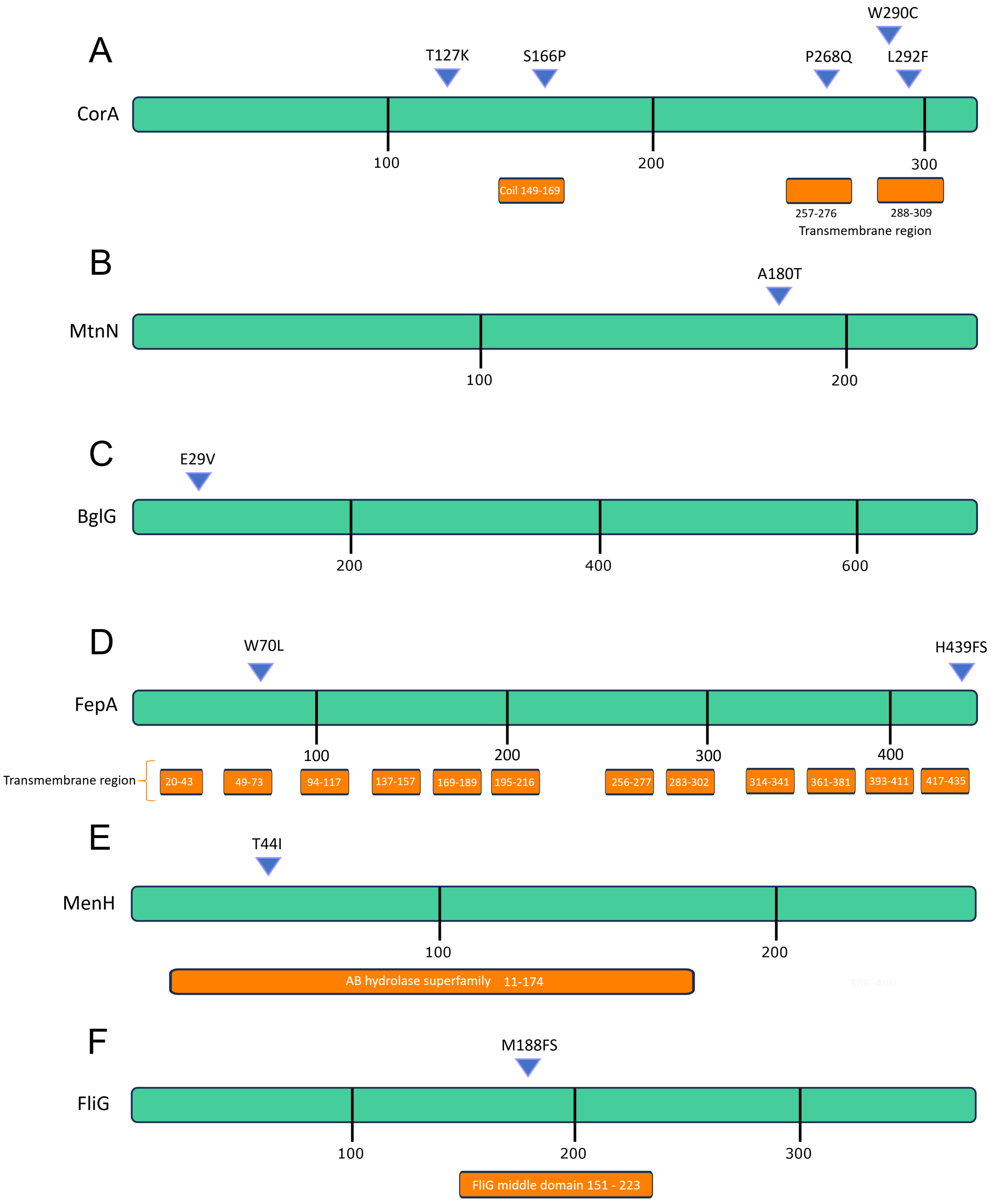

Supplementary Figure 7. Demonstrating conservation of mutations detected in YkoK leader over the course of the biofilm persistence model within (A) and outside (B) of *Listeria* genus. Demonstrated via sequence logos of nucleic acids multiple sequence alignment, consisting of 50 sequences each, which were retrieved from the NCBI database using a BLAST search. Data on each of the retrieved sequences is provided in **Supplementary Table 1** and **Supplementary Table 2**. The total stack height represents sequence conservation (information content in bits), while the relative height of each nucleotide within the stack reflects its frequency at that position. Highly conserved sites show tall stacks dominated by a single nucleotide; variable sites show shorter stacks with mixed bases. Visualised with WebLogo v2.8.2.

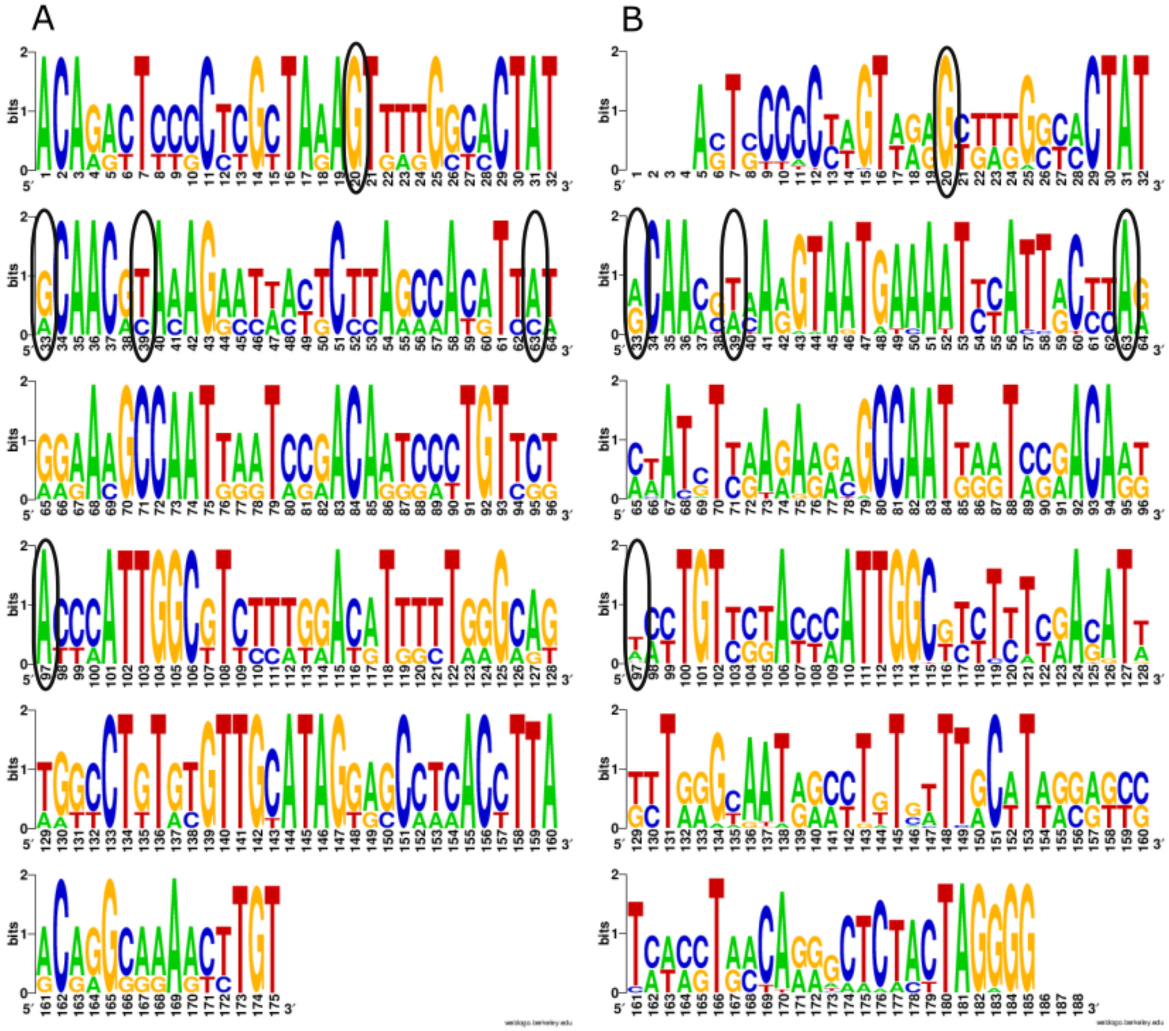

Supplementary Figure 8. Graphical overview of amino acid substitutions in (A) PitA and (B) RnfC. Non-synonymous mutations are indicated by triangle. Effected domains are demonstrated.

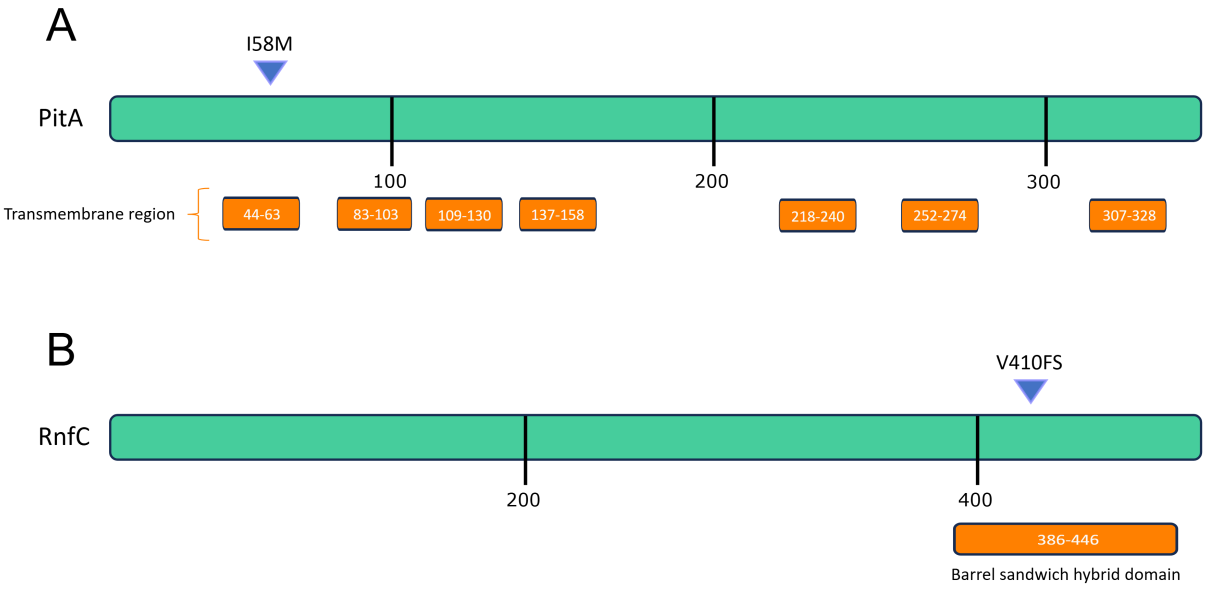

Supplementary Figure 9. Graphical overview of substitutions in SigB regulating operon for (**A; B; C**) RsbU, RsbV, RsbW. Substitutions are indicated by triangles. Predicted structural and functional domains are indicated by the orange boxes. * Indicates an early stop codon, FS – frameshift mutation. Domains predicted with InterPro.

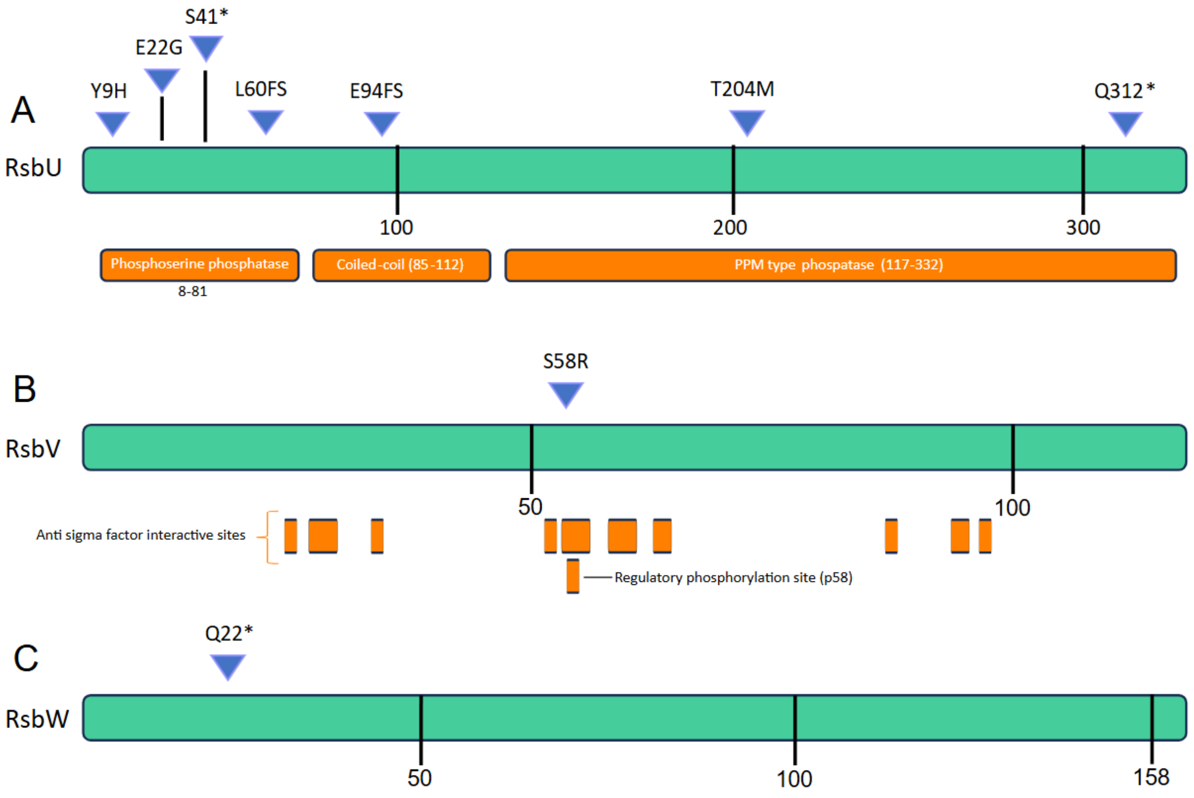

Supplementary Figure 10. Biofilm persistence did not illicit an increase in biomass/population generation. (**A**) Cell surface attachment capacity (CFU/bead) of evolved biofilm-associated lineages from late evolutionary timepoint. (**B**) Biomass production of evolved biofilm-associated lineages from late evolutionary timepoint. Includes data for BC-exposed and non-exposed groups. L1, L2, L3 indicate the number of independent lineage tested. All experiments were performed in triplicates, whiskers represent minimum to maximum values, with the median value indicated at the box plot. Every data point is demonstrated. A Kruskal-Wallis followed by Dunn's multiple comparison post hoc test was performed to compare the groups. ns = not significant.

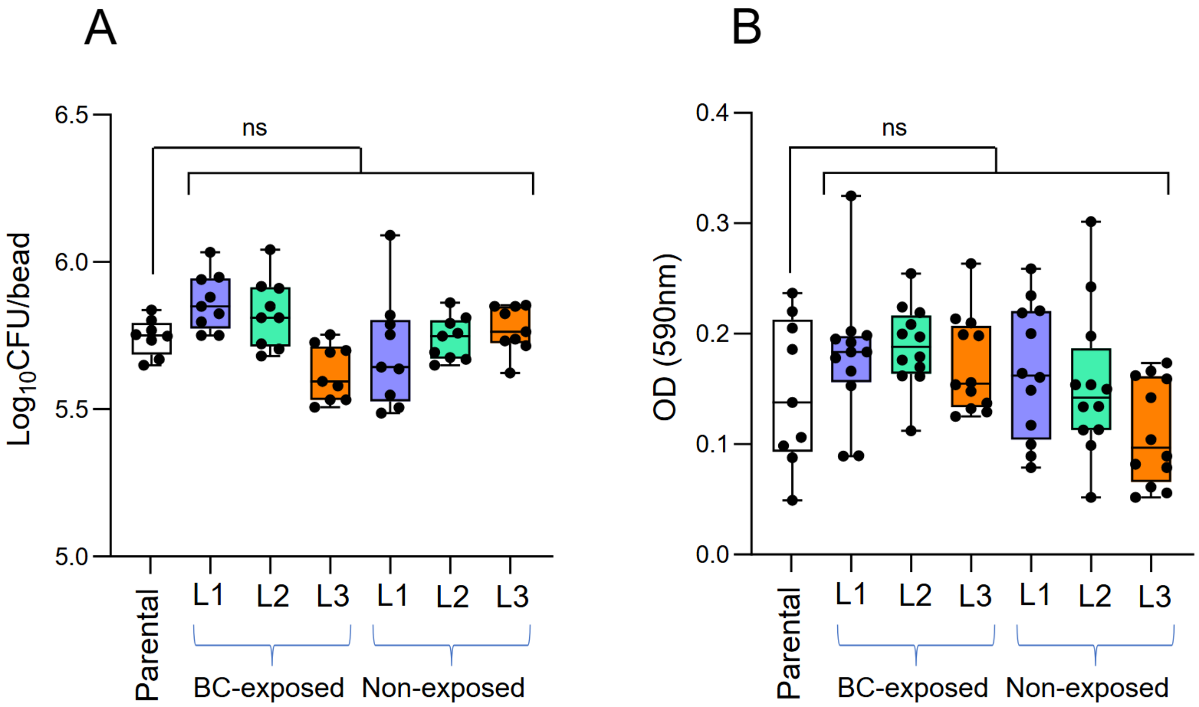

Supplement: Supplementary Material 1. [file mgen-12-01611-s001.pdf]
